# Monitoring nature's calendar from space: Emerging topics in land surface phenology and associated opportunities for science applications

**DOI:** 10.1111/gcb.16436

**Published:** 2022-09-26

**Authors:** Xuanlong Ma, Xiaolin Zhu, Qiaoyun Xie, Jiaxin Jin, Yuke Zhou, Yunpeng Luo, Yuxia Liu, Jiaqi Tian, Yuhe Zhao

**Affiliations:** ^1^ College of Earth and Environmental Sciences, Lanzhou University Lanzhou China; ^2^ Department of Land Surveying and Geo‐Informatics The Hong Kong Polytechnic University Hong Kong China; ^3^ School of Life Sciences, Faculty of Science University of Technology Sydney Sydney New South Wales Australia; ^4^ College of Hydrology and Water Resources, Hohai University Nanjing China; ^5^ Key Laboratory of Ecosystem Network Observation and Modelling Institute of Geographic Sciences and Natural Resources Research, Chinese Academy of Sciences Beijing China; ^6^ Swiss Federal Institute for Forest, Snow and Landscape Research WSL Birmensdorf Switzerland; ^7^ Department of Environmental System Science ETH Zurich Zurich Switzerland; ^8^ Geospatial Sciences Center of Excellence (GSCE) South Dakota State University Brookings South Dakota USA; ^9^ Department of Geography National University of Singapore Singapore Singapore

**Keywords:** big data, biodiversity, carbon cycle, global change, public health, vegetation dynamics

## Abstract

Vegetation phenology has been viewed as the nature's calendar and an integrative indicator of plant‐climate interactions. The correct representation of vegetation phenology is important for models to accurately simulate the exchange of carbon, water, and energy between the vegetated land surface and the atmosphere. Remote sensing has advanced the monitoring of vegetation phenology by providing spatially and temporally continuous data that together with conventional ground observations offers a unique contribution to our knowledge about the environmental impact on ecosystems as well as the ecological adaptations and feedback to global climate change. Land surface phenology (LSP) is defined as the use of satellites to monitor seasonal dynamics in vegetated land surfaces and to estimate phenological transition dates. LSP, as an interdisciplinary subject among remote sensing, ecology, and biometeorology, has undergone rapid development over the past few decades. Recent advances in sensor technologies, as well as data fusion techniques, have enabled novel phenology retrieval algorithms that refine phenology details at even higher spatiotemporal resolutions, providing new insights into ecosystem dynamics. As such, here we summarize the recent advances in LSP and the associated opportunities for science applications. We focus on the remaining challenges, promising techniques, and emerging topics that together we believe will truly form the very frontier of the global LSP research field.

## Graphical abstract

Land surface phenology (LSP), as an interdisciplinary subject among remote sensing, ecology, and biometeorology, has undergone rapid development over the past few decades. Recent advances in sensor technologies, as well as data fusion techniques, have enabled novel phenology retrieval algorithms that refine phenology details at even higher spatiotemporal resolutions, providing new insights into ecosystem dynamics. As such, we summarize the recent advances in LSP and the associated opportunities for science applications. We focus on the remaining challenges, promising techniques, and emerging topics that together we believe will truly form the very frontier of the global LSP research field.

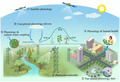

## 1 BACKGROUND

Vegetation phenology is an important and integrative proxy that characterizes the Earth system dynamics and is the key to understanding how atmosphere‐biosphere‐hydrosphere interactions respond to climate change and human activities (Fu et al., 2020; Inouye, 2022). Phenology has been a prominent diagnostic proxy as well as an input in prognostic models that is widely used in areas such as food security (Alemu & Henebry, 2016; Gao & Zhang, 2021; Gray, Friedl, et al., 2014; Lobell et al., 2008), frost hazard (Dai et al., 2013; Ge et al., 2013; Hänninen, 2006), drought (de Beurs & Henebry, 2008), forest fire risk (Bison et al., 2022), landscape dynamics, climate change (Brown et al., 2017; Friedl et al., 2014; Jeganathan et al., 2014; Jin et al., 2019), biogeochemical cycling (Gray, Frolking, et al., 2014; Piao et al., 2019). Satellite remote sensing, with its synoptic view of the Earth, has become an invaluable approach to monitoring phenology at a global scale and in a continuous and highly consistent manner (Caparros‐Santiago et al., 2021; Zeng et al., 2020).

Despite the prosperity and rapid development in the field of land surface phenology (LSP), challenges remain to be addressed and emerging new fields of application remain to be explored (Piao et al., 2019; Tang et al., 2016). From a technical perspective, inconsistent or even controversial pattern and trend in satellite phenology are often obtained with various data quality levels or retrieval algorithms, suggesting that much effort are still needed in improving the satellite phenology retrievals (Atkinson et al., 2012; Jin, Jönsson, et al., 2017; Wang, Wu, et al., 2022; Xie et al., 2022; Zheng & Zhu, 2017). Meanwhile, validation is integrated as an essential component into most LSP applications facilitated by the accessibility of established phenocam and in‐situ observation networks worldwide (Tian, Cai, Jin, et al., 2021), although scaling up from in‐situ phenology to satellite phenology observations remains a grand challenge (Peng, Zhang, Zhang, et al., 2017; Zhang et al., 2017). Besides, Internet‐of‐Things (IoT), big data, and artificial intelligence (AI) are being increasingly adopted in phenology studies. IoT can generate massive amounts of data streamed from cameras, phenology sensors, or even social medias, so called “pan‐spatial data” (Zhou et al., 2022). The new data from IoT demand non‐conventional analytic approaches such as text mining, computer vision, and AI that can truly take advantage of the pan‐spatial data and further offer a complementary view of global phenology pattern to satellite observations.

From a scientific perspective, the breadth of LSP applications is expanding to an even great and more diverse extent. Phenology has traditionally been considered the key to understanding carbon–water coupling (Fu et al., 2020), yet a quantitative and mechanical understanding has not been achieved. Meanwhile, how climate factors affect phenology has been studied extensively over the past decade, and very recently factors beyond climate such as nitrogen deposition have started gaining attention (Luo et al., 2020; Wang et al., 2020). In addition, LSP has also been integrated into the early‐warning system for pollen outbreak forecast, which is highly relevant to public health (Devadas et al., 2018; Li et al., 2019). In this case, a multidisciplinary approach that integrates ecologists, meteorologists, epidemiologists, and remote sensing scientists is required.

**FIGURE 1:**
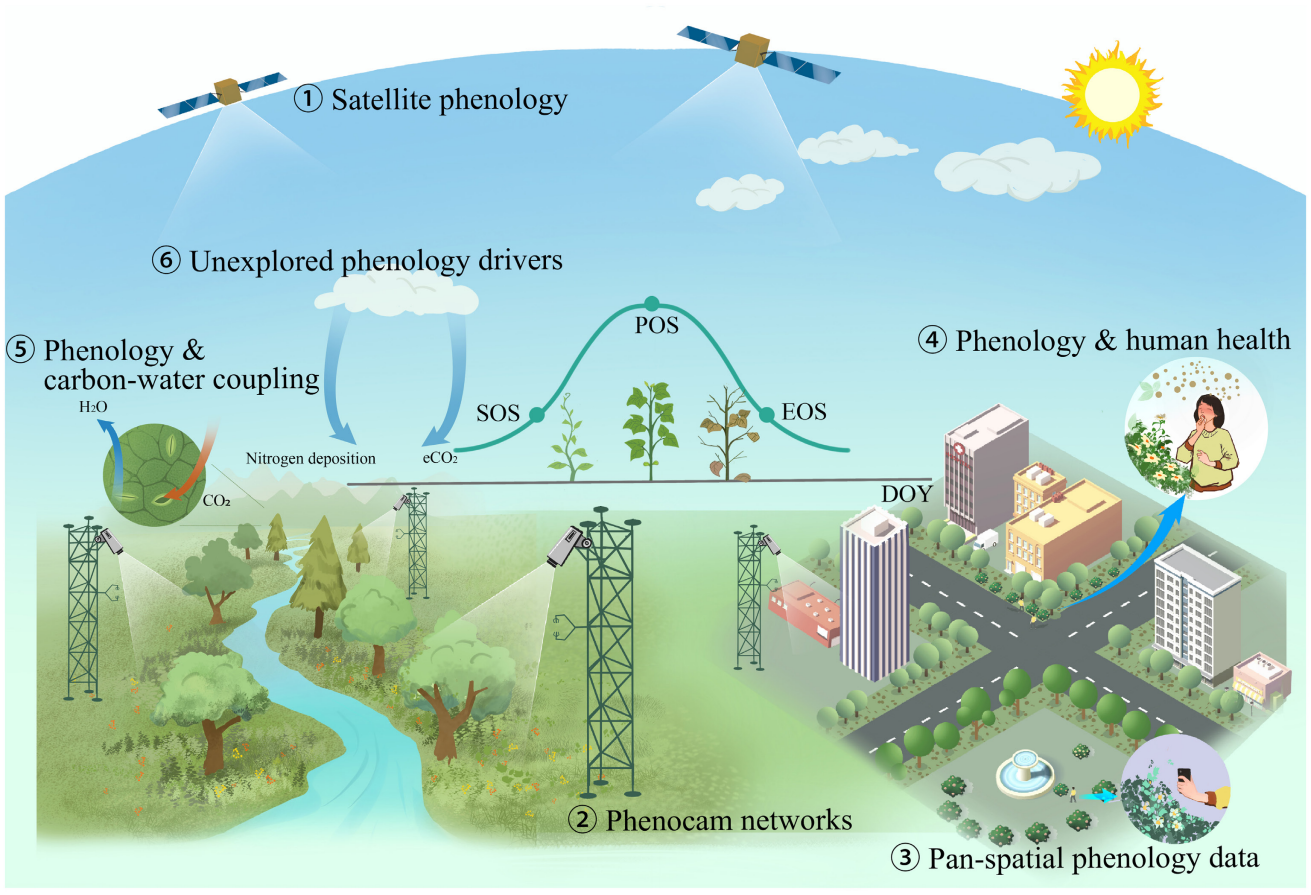
Overview of the six emerging topics discussed in this review article.

In this context, here we provide a review on the emerging topics that are either related to the scientific applications or the technical issues of LSP. We noted recent reviews on remote sensing phenology retrieval methodologies (e.g., Zeng et al., 2020) as well as phenology and climate change (e.g., Piao et al., 2019). Our specific review, therefore, focused more on the selected topics to highlight opportunities to advance the research frontier instead of repeating what has been covered in previous review articles. Figure 1 provides a graphical overview of the six topics we discussed in this article. The first three topics focused on the technical aspects of LSP, followed by three topics focusing on the rising opportunities for phenology‐related science applications.

## 2 UNCERTAINTIES IN LAND SURFACE PHENOLOGY

**FIGURE 2:**
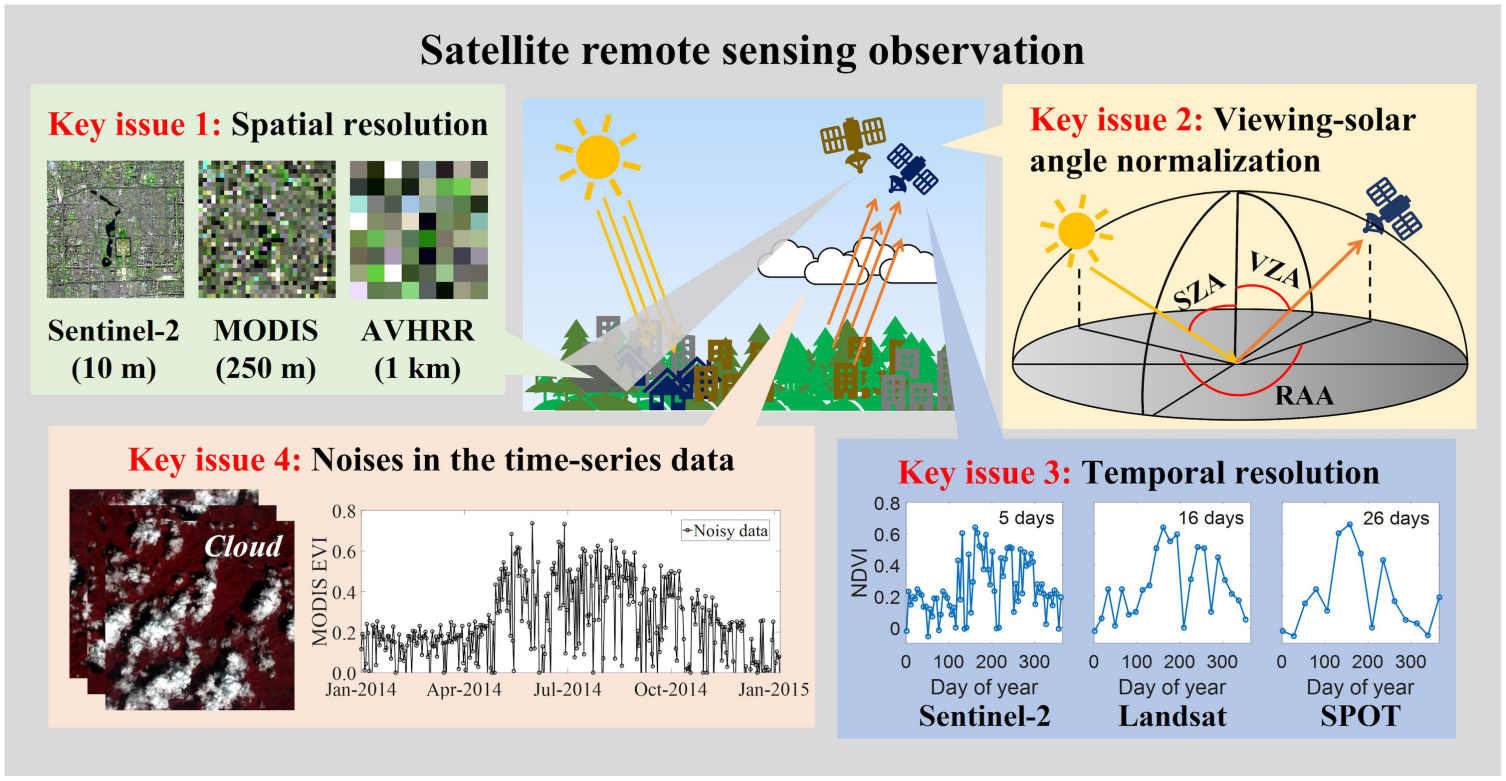
Diagram of key factors related to the uncertainties (i.e., spatial resolution, temporal resolution, viewing‐solar angle normalization, and noises in the time‐series data) in land surface phenology using satellite‐based remote sensing observation. RAA, relative azimuth angle; SZA, solar zenith angle; VZA, viewing zenith angle.

Existing studies often obtain inconsistent or controversial results even on the same research question (Han & Xu, 2013; Qiu et al., 2017), suggesting large uncertainties in phenology metrics derived from satellite observations, and even official phenology products. For instance, validated with ground PhenoCam observations, root mean square error (RMSE) values of the start of the season (SOS) and the end of the season (EOS) are 12.3 and 21.3 days for VIIRS LSP product, and 10.1 and 21.6 days for MODIS land cover dynamics product respectively (Moon et al., 2021). The intercomparison of six phenology products (i.e., MCD12Q2, VIPPHENEVI2, CMGLSP, MOD09Q1PEVI, MOD15PHN, and AVHRRP) showed that RMSE of SOS retrievals of these phenology products are around 20 days (Peng, Zhang, Wu, et al., 2017). The differences in vegetation phenology detection among diverse satellite‐based phenology products may result from the uncertainties following major factors (Figure 2), including spatial resolution (i.e., mixed pixel effect caused by coarse spatial resolutions), sun‐view geometry effect, temporal resolution (i.e., low‐frequency observations), and noises (i.e., atmospheric effects including clouds, hazes, and aerosols). Here we summarize and discuss recent studies on the above four factors, which could help future studies to improve the reliability of satellite‐based vegetation phenology detection.

First, satellite images of various spatial resolutions from 10 m to 1 km were used to extract vegetation phenology (Cong et al., 2012; Melaas et al., 2013; Shen et al., 2014; Tian, Zhu, Shen, et al., 2020; Zhang et al., 2003). Because of the scale effects (Chen et al., 2018; Peng, Zhang, Zhang, et al., 2017; Zhang et al., 2017), the coarse‐resolution images cannot always provide vegetation phenology accurately at the desired spatial scales, resulting in the misestimation of vegetation phenology, especially in the fragmented vegetation regions (Zhu & Liu, 2019). For example, Qiu et al. (2017) reported the average rural–urban difference of spring phenology was on the order of 5–10 days using 30‐m Landsat data, but it was 7–15 days when using 1‐km SPOT data in the same study area (Han & Xu, 2013). Similarly, the average rural–urban difference of green‐up dates in Salt Lake City, United States, was more than half a month using 500‐m MODIS data (Li et al., 2016), whereas it was less than 4 days when using 30‐m fused data (Gervais et al., 2017). A recent study reveals that coarse‐resolution satellite images would overestimate the rural–urban difference in phenological metrics (Tian, Zhu, Wu, et al., 2020). A potential reason for this overestimation is that the diversity of spring phenological dates is greater in coarser urban pixels, thus causing spring phenological dates extracted from coarser satellite images to be generally earlier than actual dates, which is agreed with another two recent papers revealing that spring phenology derived from coarse satellite images can be more controlled by vegetation species with earlier spring phenology (Chen et al., 2018; Liu et al., 2019).

Second, the necessity of BRDF adjustment including satellite viewing angle and solar illumination angle for phenology detection is broadly documented. For example, a recent study indicated that satellite viewing angles greatly increased the uncertainty of vegetation phenology extraction (Lu et al., 2022). Moreover, the seasonal changes in solar zenith angle (SZA) can also alter the temporal trajectory of the VI time series, thereby causing a lower precision of vegetation phenology extraction than that of fixed SZA (Ma et al., 2019, 2020; Norris & Walker, 2020). As a result, to acquire more accurate results of phenological metrics, the BRDF normalization should be implemented before vegetation phenology extraction (Morton et al., 2014; Petri & Galvão, 2019). Fortunately, some official satellite products have offered the opportunity for correcting the BRDF effects, for example, MODIS MCD43A1 BRDF/Albedo Model Parameters (Schaaf et al., 2002) and Harmonized Landsat and Sentinel‐2 (HLS) surface reflectance products (Claverie et al., 2018).

Third, satellite data with sparse temporal resolutions (e.g., Landsat 16 days) may not be capable of capturing the key stages of vegetation phenology, resulting in higher uncertainty in phenology retrieval and reduced ability in detecting inter‐annual variability or long‐term trend. A simulation study based on MODIS data shows that vegetation phenology can be detected with satisfying precision (absolute errors are less than 3 days) with temporal resolutions up to 16 days (Zhang et al., 2009). Another recent study used simulated EVI with daily to 52 days temporal resolutions to detect spring phenology in North America and found that temporal resolutions nonlinearly affected the accuracy of LSP (Tian, Zhu, Wan, et al., 2021).

Fourth, time‐series smoothing (e.g., maximum value composite and temporal filters) is a conventional step to process daily noisy satellite data (Cai et al., 2017; Chen et al., 2004). The arbitrary choice of smoothing methods and parameters may affect the precision of phenology detection considering that cloud covers have high spatial heterogeneity (Ju & Roy, 2008; Wilson & Jetz, 2016). For example, a study uncovered that the spring phenology derived from the coarse composites was earlier than that derived from the fine composites (Zhu et al., 2019). A recent study investigated the impact of clouds on the smoothing process at a global scale and recommended optimal smoothing parameters for future studies in different regions (Tian, Zhu, Chen, et al., 2021).

To address the issues of the above potential uncertainties in LSP using satellite‐based remote sensing observation. some cutting‐edge image reconstruction technologies can be used to optimize the spatial and temporal resolutions and reduce noises in time‐series data, for example, cloud and gap‐filling technologies (Zhu et al., 2021) and data fusion technologies (Tian, Zhu, Wu, et al., 2020). Alternatively, new generation geostationary satellite (e.g., Advanced Baseline Imager, ABI) images and CubeSat constellation (e.g., PlanetScope) images provide high‐frequency and fine‐resolution observations which can further alleviate the uncertainty effects. In addition, except the uncertainties mentioned above, the choice of different satellite data sets (e.g., AVHRR and MODIS), VI time‐series data (e.g., NDVI and EVI), and phenology extraction algorithms (i.e., threshold‐based and curvature‐based methods) also may result in the uncertainty of detection results, but the difference is slight for these factors (Cong et al., 2012; Shen et al., 2014). It is worth noting that solving the abovementioned uncertainties could mainly improve landscape‐scale phenology detection using satellite remote sensing observations, which may not work for phenology detection from the individual tree to leaf scales. To address this issue, a possible solution is to integrate multiscale observations from the space, sky, and ground. This is also a new perspective and a frontier for future vegetation phenology studies.

## 3 PHENOCAM TRACKING FINE‐SCALE ECOSYSTEM DYNAMICS AND MECHANISM

Understanding how phenology responds to environmental change globally and validating satellite phenology products require more high‐quality field‐collected data (Berra & Gaulton, 2021; Brown et al., 2016; Richardson, Hufkens, Milliman, Aubrecht, Chen, et al., 2018). The traditional method of field observations for phenology states is based on human observers (Li, Shen, et al., 2021). Although this approach provides species‐specific ground phenology observations (Klosterman et al., 2014), inherent subjectivity, inconsistency in temporal resolution, and insufficient spatial representativeness restrict the field records to characterize vegetation phenology at the regional scale (Richardson, Hufkens, Milliman, Aubrecht, Chen, et al., 2018). Furthermore, direct phenological surveys provide observations with fine biological details and representation of diversity, but they cannot well represent the phenological response of the whole community (Berra & Gaulton, 2021). ‘Near‐surface’ remote sensing—phenocam integrates phenological signals across the whole vegetation canopy, offering opportunity for satellite validation on the one hand, and distinguishing individual plant phenology on the other hand (e.g., single tree crowns) (Seyednasrollah et al., 2019).

Phenocam represents any digital camera used for automatic time‐lapse photography to observe the variations in the vegetation cover continuously at a high temporal frequency and spatial resolution (Brown et al., 2016; Richardson et al., 2013). Large‐scale phenocam networks have been developed and widely adopted for monitoring ecosystem dynamics worldwide within the last decade (Berra & Gaulton, 2021; Richardson, Hufkens, Milliman, Aubrecht, Chen, et al., 2018). For example, the PhenoCam network (http://phenocam.sr.unh.edu; Richardson, Hufkens, Milliman, Aubrecht, Chen, et al., 2018) and Phenological Eyes Network (PEN; http://pen.agbi.tsukuba.ac.jp) (Nasahara & Nagai, 2015). The US National Ecological Observatory Network (NEON) (Utz & Prism, 2012) and the European Union's Integrated Carbon Observation System (ICOS) (http://european‐webcam‐network.net/) have also established phenocam networks. In the Southern Hemisphere, the Australian Phenocam Network (APN) (https://phenocam.org.au) was established to facilitate the sharing of phenocam data and researches using this novel technology and to obtain a better understanding of phenological dynamics in this continent (Brown et al., 2016; Marchin et al., 2018; Moore et al., 2016). Crucial issues related to data standardization, open‐access data, and expanding phenocam networks are important for the development of phenocam technology (Richardson, Hufkens, Milliman, Aubrecht, Chen, et al., 2018).

**FIGURE 3:**
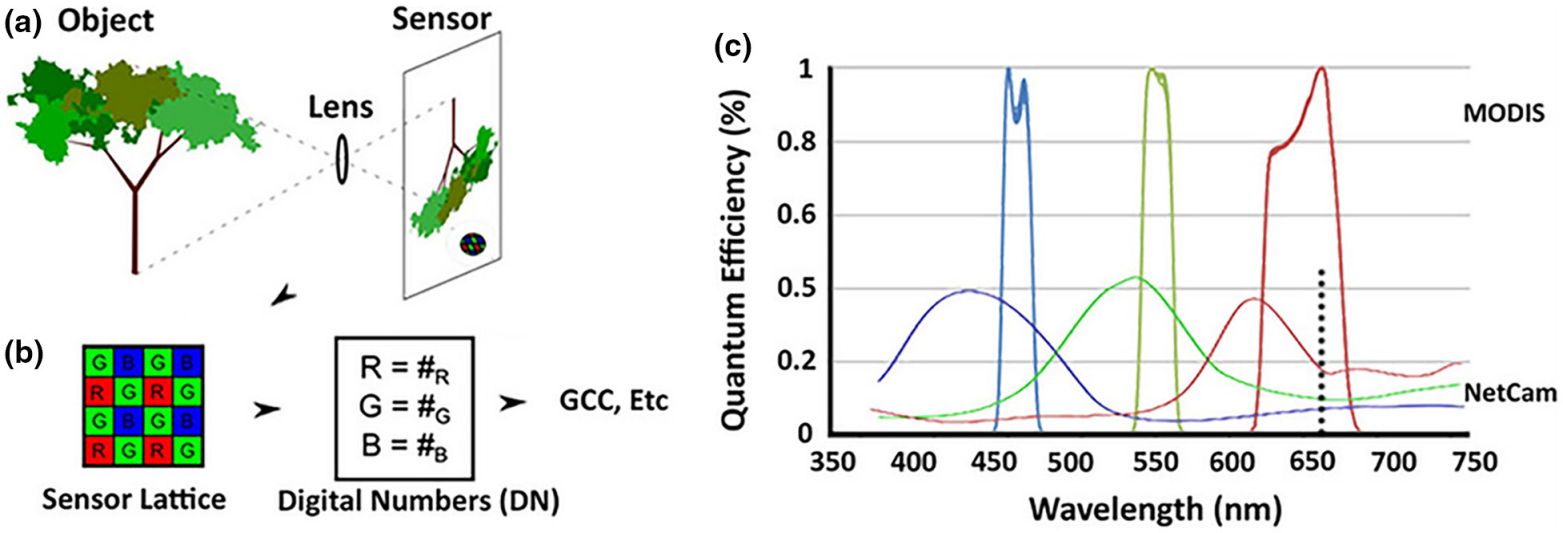
Diagram shows how phenocam work (a and b) and typical spectral response for MODIS and camera imaging sensors (c). Source: Brown et al. (2016).

Technologically, the camera lens projects the observed object onto a digital chip with a light‐sensitive structure. Three channels (RGB) are used to record the color information of the landscape. However, RGB digital number values are generally not directly used to conduct phenological analysis due to both external and internal factors—scene illumination affected by clouds, aerosols, solar azimuth, and so on, and control of exposure and color balance adjustment—could bring uncontrollability in image processing (Sonnentag et al., 2012). To better track phenology variability, the respective chromatic coordinates of RGB DN values (RCC, GCC, and BCC) are widely used in phenology studies (Brown et al., 2016; Klosterman et al., 2014; Liu, Fu, et al., 2016; Migliavacca et al., 2011; Moon et al., 2022; O'Connell & Alber, 2016; Songsom et al., 2021). Other color indices have since been construed by the nonlinear transformation of RGB DN values, including the VARI (Visible Atmospherically Resistant Index) (Sakamoto et al., 2012), grR (green‐red ratio) (Sonnentag et al., 2011), as well as the excess green (ExG) (Woebbecke et al., 1995). Furthermore, Figure 3 shows how camera sensors work (a and b), and the spectral responses of MODIS and imaging sensors (c) (Brown et al., 2016). Typical phenocam records overlapping RGB bands, with the near‐infrared and part of the red region beyond about 650 nm are often omitted in most commercial cameras (dotted line in Figure 3c). The MODIS sensors, however, have no band overlapping and higher sensitivity to specific wavelengths compared with commercial cameras (Brown et al., 2016). Although phenocam image processing technology has made great progress, the inherently spectral limitations of camera sensors should not be overlooked for vegetation phenology detections, especially ground validation for satellite‐derived phenology observations.

Phenocam has become a promising way for phenological studies in forests, grasslands, and agricultural areas (Baumann et al., 2017; Brown et al., 2017; Keenan et al., 2014; Khare et al., 2021; Nietupski et al., 2021; Ren & Peichl, 2021; Richardson et al., 2012; Song et al., 2022; Tian, Cai, Jin, et al., 2021; Toomey et al., 2015). Additionally, phenocam images have been used to derive important information on snowmelt processes (Kim et al., 2021; Zheng et al., 2022). Using the phenocam imagery and satellite data, Liu et al. (2017) evaluated the detection of vegetation phenology in savannas and grasslands. Their results showed that the phenocam NDVI was strongly correlated with the satellite NDVI for two grassland ecosystems. Recently, a study using HLS, PlanetScope data, and phenocam imagery reported that not only the VI temporal profiles from satellites and phenocam show high temporal agreement but also phenometrics derived from all three data sets agreed very well with each other (Moon et al., 2021).

Phenocam color indices are well correlated with satellite VIs typically used to detect LSP, suggesting that phenocam can provide good ground measurement data for verifying satellite phenology detections (Liu et al., 2021; Thapa et al., 2021; Zhang et al., 2018). For example, researchers used phenocam observation as the ground validation for a new algorithm of 30 m LSP product derived from HLS and VIIRS surface reflectance products (Zhang et al., 2020). Phenocam data are also combined with other ecological observation data, such as surface‐atmosphere fluxes, to characterize the responses of vegetation productivity to phenological variations (Browning et al., 2021; Wingate et al., 2015) and thus depict the relationships between seasonal plant dynamics and ecosystem carbon budgets (Vázquez‐Lule & Vargas, 2021). Through combined phenocam technology with eddy covariance data at a subalpine grassland, digital camera imagery was demonstrated to have the potential for the parameterization of phenological and radiation use efficiency models (Migliavacca et al., 2011). Combining carbon flux data from FLUXNET2015 Dataset (https://fluxnet.org/data/fluxnet2015‐dataset/), PhenoCam, and MODIS, researchers reported the summer physiology can explain the interannual variability of NEP (net ecosystem productivity) for most ecosystems, besides grassland. This finding highlights the significance of understanding the role of summer physiology in carbon accumulation (Liu & Wu, 2020). More recently, Moon et al. (2022) used PhenoCam data as ground reference to present a high spatial resolution (3 m) LSP data set for AmeriFlux and NEON sites across North America using PlanetScope imagery.

In addition to validating LSP derived from satellites and coordinating with flux data to interpret carbon sequestration of terrestrial ecosystems, multispectral cameras on board unmanned aerial vehicles (UAVs) are increasingly applied to depict plant characteristics, for example, leaf area index (LAI), texture, and plant height (PH), especially in agricultural application scenarios (Hassan et al., 2019; Su et al., 2018; Zhou et al., 2017). Using a multi‐spectral camera attached to a UAV, researchers assessed the capability of vegetation indices calculated from cameras to capture variations in LAI and plant counts, which are of interest to sorghum breeders, and consequently to inform the sorghum breeding practice (Potgieter et al., 2017). Shu et al. (2022) improved the accuracy of UAV‐based digital imagery (RGB bands) in monitoring maize aboveground biomass by integrating PH and LAI predicted from UAV‐based multi‐spectral images. Using ground truth flowering data derived from the UAV‐based RGB images to label flowering pixels in PlanetScope images, Dixon et al. (2021) produced a landscape‐scale flowering phenology map for Southwest Australia eucalypt canopies.

## 4 PAN‐SPATIAL BIG DATA AND SMART SENSING FOR LAND SURFACE PHENOLOGY

**FIGURE 4:**
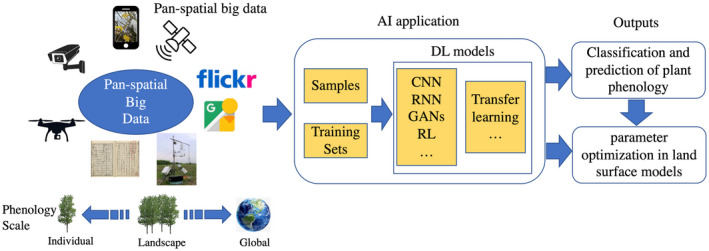
Workflow for pan‐spatial big data, smart sensing, and AI applications in plants phenology analysis.

There has been a long history of artificial observation of plant phenology, which mainly aims to adapt to climatic changes and improve agricultural management activities (sowing, harvesting, etc.; McGowan et al., 2021; Ren et al., 2019; Vitasse et al., 2022). In many countries, ancient literary works and agricultural books have recorded abundant clues in plant phenology (e.g., tree flowering, bird migration) and climate changes (e.g., snowing in low‐latitude regions) (Dye, 2002; Shi et al., 2017; Vitasse et al., 2022). From the current perspective, these observations naturally have a citizen science‐like characteristic implying phenological observations can be performed by everyone. Nowadays, millions of automatic sensing equipment have been deployed over the Earth's surface and around space, such as web cameras, weather (ecological) stations, satellites, and drones, to monitor land surface changes. The wide use of portable smart devices (e.g., cell phones, cameras mounted in cars) and their data sharing through internet, also have greatly improved the information capacity of the Earth's observation database. Thus, a huge sensing data flow with the spatial feature, from real or cyber space, constructs a multivariate data pool that together can be termed as “pan‐spatial big data” (Figure 4; Zhou et al., 2022). Therefore, we will enter into a big data era for phenological studies that on the one hand forms invaluable data for comparative and integrative spatial phenology analysis with satellite remote sensing, whereas on the other hand demands technical advances in various dimensions, such as data quality filtering and data mining approach.

Currently, AI technique has demonstrated a promising performance in extracting information from pan‐spatial big data (Boukabara et al., 2021; Irrgang et al., 2021; Mehajan & Verma, 2020; Sun et al., 2022). However, researchers need to identify specific areas where AI techniques could be used for phenological study across multiple scales, that is, from individual plant to landscape phenology, as well as from texts, photos, to other information acquired with a variety of new sensors (Figure 4). Massive street‐level imagery has been applied to monitor crop phenology based on deep learning (DL) and unleashed its power in plant classification and phenology identification (d'Andrimont et al., 2022; Hufkens et al., 2019). Using computer vision and machine learning methods, plant individual phenology (e.g., bud bursting, leafing) can be detected from noisy time‐lapse images (Correia et al., 2020). Plant growing data collected by volunteers have increasingly contributed to mapping phenological status, jointly used with remotely sensed data (Elmore et al., 2016; MacKenzie et al., 2017; Wallace et al., 2016), whereas data quality should be carefully assessed to be consistent with scientific intentions. Crowd‐sourced photographs from social sensors (e.g., Flickr, Twitter) also construct a geospatial cloud for monitoring phenology (Breckheimer et al., 2019; Cope et al., 2017). For instance, a smartphone application was designed to record bud bursts. Text mining technique was also used for revealing autumn phenology information from social networking platforms such as Twitter, Flicker, and Weibo (Nagai et al., 2021). It should be noted that there is much to improve in terms of the accessibility of pan‐spatial phenology data. For instance, although a significant amount of urban photos are taken by the surveillance camera that has a potential for retrieving phenology, but most of these data are restricted from access. Besides, it is also time‐consuming for the researchers to retrieve phenology information from internet sources. From this perspective, an open‐source repository of pan‐spatial phenology data with standardized format and metadata would surely be valuable to the community.

The big challenge for AI application in phenological pan‐spatial data will exist in its entire workflow, including data pre‐processing, model training, and evaluation. How to obtain a statistically meaningful sample density for training, which will greatly impact its applicability, as well as the credibility of results. For instance, if we use DL approach to identify tree flowering dates based on photos retrieved from web‐camera or cell phones, the reliability of the result relies on the specie classification accuracy of flower images. Although the classification of plants and their phenological phase from imagery depends on human interpretation of sample images, which highly needs professional knowledge. In that case, it will reduce the cost‐efficiency in AI‐based phenological analysis and introduce some uncertainties in model outputs. Additionally, it is difficult to construct an efficient and widely applicable deep neural network in AI phenological experiments. Even though there are many DL networks in image analysis, transfer learning from these models requires great programming skills to adapt model parameters. Lastly, the optimal workflow to scale up from pan‐spatial phenology to regional or even global scale, in conjunction with LSP remains to be established.

## 5 LAND SURFACE PHENOLOGY AND HUMAN HEALTH

Satellite phenology has been used in human health‐related science applications. Studies have reported shifts in forest and grass phenology associated with changing climate (Buermann et al., 2013; Munson & Long, 2017; Xie et al., 2022), which could contribute to changing allergenic pollens exposure due to altered flowering and pollination times. Pollen exposure is projected to intensify with climate change and changes in land cover, raising the risks for allergic respiratory diseases that pose threats of severe public health problems (Rojo et al., 2015). This suggests more days with high pollen concentration and more extreme events like thunderstorm asthma. These diseases afflict nearly 500 million people worldwide (Khwarahm et al., 2017). Pollen concentration forecast is important to help public health emergency planning and response arrangements around events like thunderstorm asthma. A major shortcoming in current pollen surveillance methods is that they do not include available ecological information on plant species composition and plant phenology, land cover conditions (McInnes et al., 2017), and spatially detailed information on pollen concentration.

The amount of pollen in the atmosphere at any given location depends on many factors, including the vegetation type and vegetation coverage in the area, climate factors, and geographical conditions. Estimation of allergic pollen has been done using these factors together with patients' symptom reports and local expert knowledge (Silver et al., 2020) through linear and nonlinear regression models (Smith & Emberlin, 2006). These traditional approaches using statistical‐based receptor‐orientated models (Skjøth et al., 2010) are observation‐based. They usually use multiple years of pollen concentrations (Sánchez et al., 2007), chilling requirements and photoperiod process models (García‐Mozo et al., 2009), or meteorological data‐driven models (Voukantsis et al., 2010). However, forecast models based on empirical relationships between these factors with airborne pollen concentrations from one site are not likely to be suitable for other locations in different environments.

**FIGURE 5:**
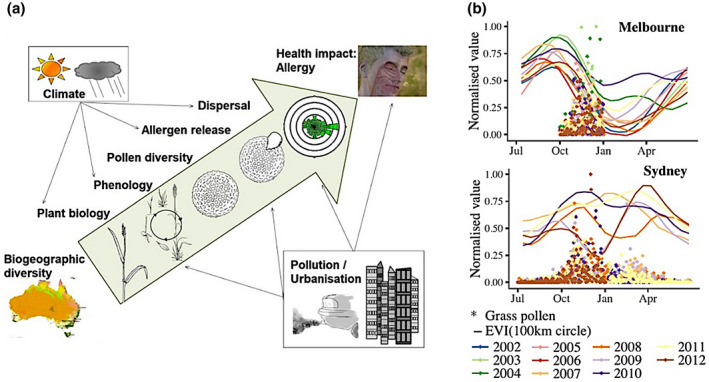
(a) Release, dispersal and impact of pollen on respiratory health (Davies et al., 2015); (b) normalized pollen concentration at monitoring sites and normalized satellite‐derived Enhanced Vegetation Index within 100 km to pollen monitoring sites at Melbourne and Sydney in Australia (Devadas et al., 2018).

Plant phenology information is critical to decipher climate and ecological‐driven factors of pollen aerobiology, and such information should aid in the short‐term pollen concentration forecasting as well as future trends of pollen aerobiology, as shown in Figure 5a (Davies et al., 2015). Moreover, pollen emission and transport have not been well studied (Emmerson et al., 2019), due to a lack of emission inventories of the pollen‐producing species, for example, the distribution and abundance, within a given geographical area. Research progress has been made on tracking pollen sources at large scales mostly in Europe (Bogawski et al., 2019; Skjøth et al., 2010; Thibaudon et al., 2014); however, the change in the vegetation cover associated with global warming requires dynamic monitoring of such pollen sources. Pollen forecasts have been achieved mostly at local scales so far and rely on statistical relationships between pollen and meteorological factors, or labor‐intensive pollen monitoring traps that are only available at limited sampling locations (Devadas et al., 2018).

Recently there is an increase in using remote sensing derived LSP data to expand the restrictive coverage of in‐situ pollen networks. The phenological timing when grass turns from maximum greenness to a drying, flowering period, and season peak was used in pollen monitoring (Emmerson et al., 2019). Devadas et al. (2018) have found close relationships between strongly seasonal and pronounced pollen periods and satellite‐derived greenness (Figure 5b), which shows the power of using satellite remote sensing data to spatially extend point‐based pollen forecasts. However, remote sensing pollen surveillance studies have so far mostly been carried out in Europe and the United States only (Khwarahm et al., 2017; Skjøth et al., 2013) and the distribution areas of the important allergenic pollen types are mapped at a regional scale. To improve the capacity and accuracy of pollen forecast, satellite‐derived vegetation phenology should be incorporated to track the up‐to‐date composition and biogeographical distribution of species and their seasonal timings (Campbell et al., 2020; Davies et al., 2021). This ecological information will provide insights into patterns of pollen release and distribution and prediction of future pollen outbreaks (Huete et al., 2019).

Advances in satellite monitoring capabilities, phenology research, and machine learning models now make it feasible to develop and implement pollen exposure observation and forecast in both urban and regional areas. By tracking all the key stages in grass pollen production through pollen release and dispersal, the improved pollen forecast models could enhance our understanding of environmental drivers of allergic respiratory disease as well as mitigating human health threats.

## 6 LAND SURFACE PHENOLOGY AND CARBON–WATER COUPLING

**FIGURE 6:**
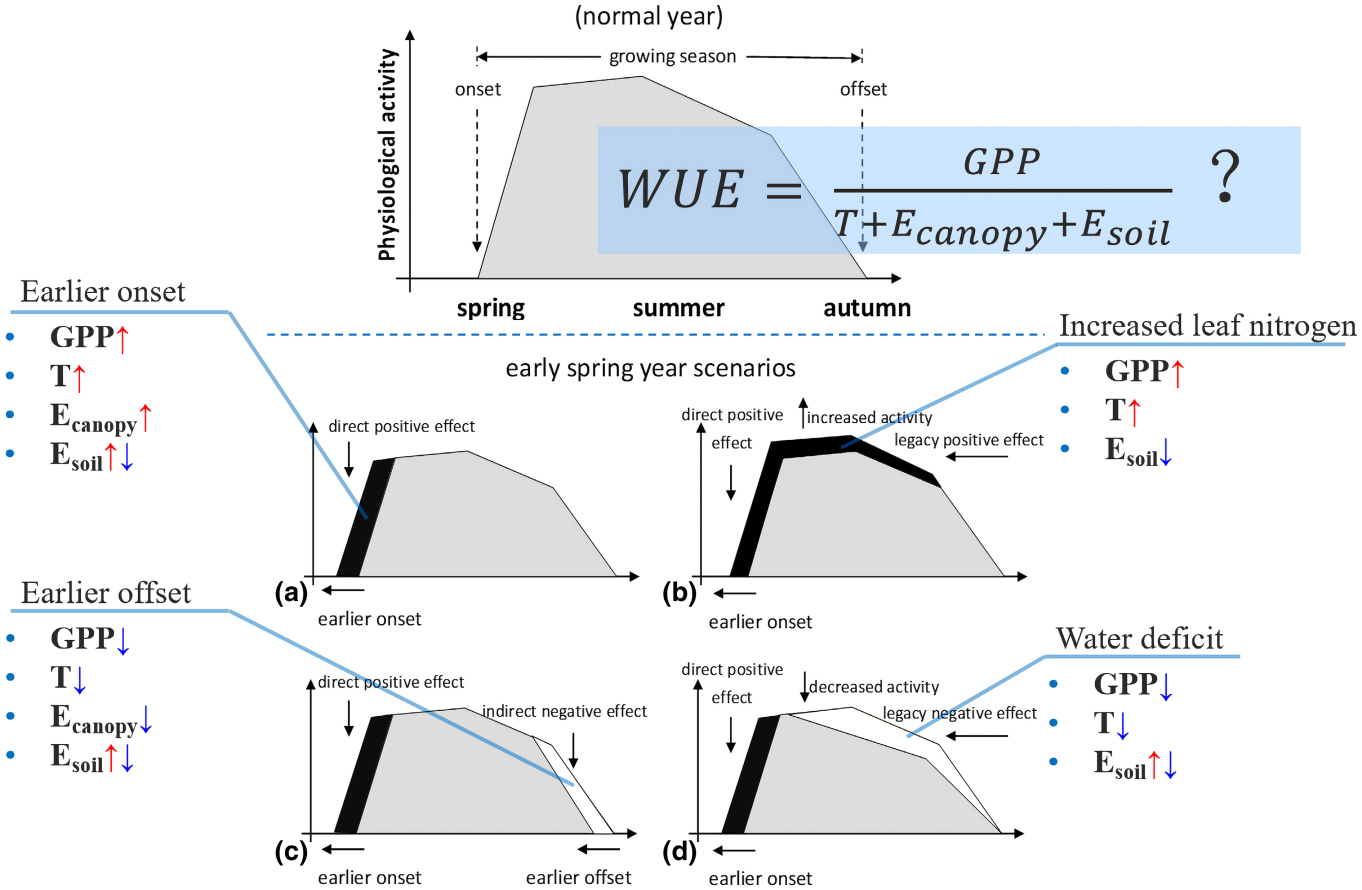
The conceptual scenarios of water use efficiency in response to spring phenology, which follows Richardson et al. (2010). *E*_canopy_, canopy interception evaporation; EOS, end of the season; *E*_soil_, soil evaporation; GPP, gross primary productivity; SOS, start of the season; *T*, transpiration

LSP and other surface variables derived from satellite observations have been adopted extensively in exploring the relationship between phenology and carbon–water coupling. From the individual plant level to ecosystem level, phenological variabilities can alter physiological and structural traits, including photosynthetic rate/light use efficiency, stomatal/canopy conductance, LAI and surface roughness, etc. (Keenan et al., 2014; Piao et al., 2007; Richardson et al., 2010, 2013; Shen et al., 2014; Wu et al., 2013). Hence, phenology directly/indirectly, positively/negatively, and synchronously/asynchronously regulates carbon (e.g., photosynthesis and respiration) and water (e.g., water absorption and evapotranspiration) exchanges on the land surface. Here, the coupling between carbon gain and water loss in response to phenology is a notable topic. Water use efficiency (WUE) is calculated as the ratio of carbon assimilation per unit of water consumption. WUE is a crucial ecological indicator, that is, the coupling capability between carbon and water cycles (Keenan et al., 2013; Tang et al., 2014). Given the difference in sensitivities of photosynthesis and transpiration to variation in phenology, the responses of WUE to phenology can potentially vary with the magnitude of the coupling between them (Richardson et al., 2013). For example, the conceptual scenarios shown in Figure 6, which follow Richardson et al. (2010), exhibit diverse variabilities in the components of WUE in response to an earlier spring phenology.

In spring and autumn, ecosystem‐scale WUE is closely associated with the satellite phenology, for example, SOS and EOS of the growing season, across the continents (Jin, Wang, et al., 2017). The variability of WUE to SOS and EOS could be explained by the difference in sensitivities of carbon gain and water loss to the phenological indicators. For example, an increasing spring (or autumn) WUE with an advanced SOS (or delayed EOS) might because the magnitude of enhanced GPP is larger than that of simulated ET, or an increase in GPP is accompanied by a decrease in ET with an earlier SOS (or a later EOS) (Beer et al., 2009; Keenan et al., 2014; Kljun et al., 2006; Luyssaert et al., 2007; Zha et al., 2010). However, summer WUE was less related to or slightly reduced by SOS due to water deficit and/or plant ecological strategy (Leuzinger et al., 2005; Wolf et al., 2016). In the Northern Hemisphere, the sensitivity of WUE to phenology exhibits a gradual enhancement from warm to cold climates. Specifically, the sensitivity of spring WUE to SOS showed a significantly negative correlation with radiation, which was associated with dramatic water loss in the high radiation part; the sensitivity of WUE to SOS in summer increased along the precipitation gradient while decreased along the temperature gradient. This might be resulted from the compensation of GPP to the delayed SOS and water deficiency due to heat stress. The sensitivity of autumn WUE to EOS enhanced significantly with both radiation and precipitation, which may be attributed to the increase of energy and water for photosynthesis. Despite the variance of the sensitivities for different PFTs in homogeneous climatic conditions, the degree of variation is much less than that in heterogeneous climates, showing a fundamental similarity of ecological functions over a broad spectrum of climates, with the respective characteristics of different plant types (Jin, Zhan, et al., 2017).

## 7 UNEXPLORED DRIVERS OF PLANT PHENOLOGY: BEYOND CLIMATE

**FIGURE 7:**
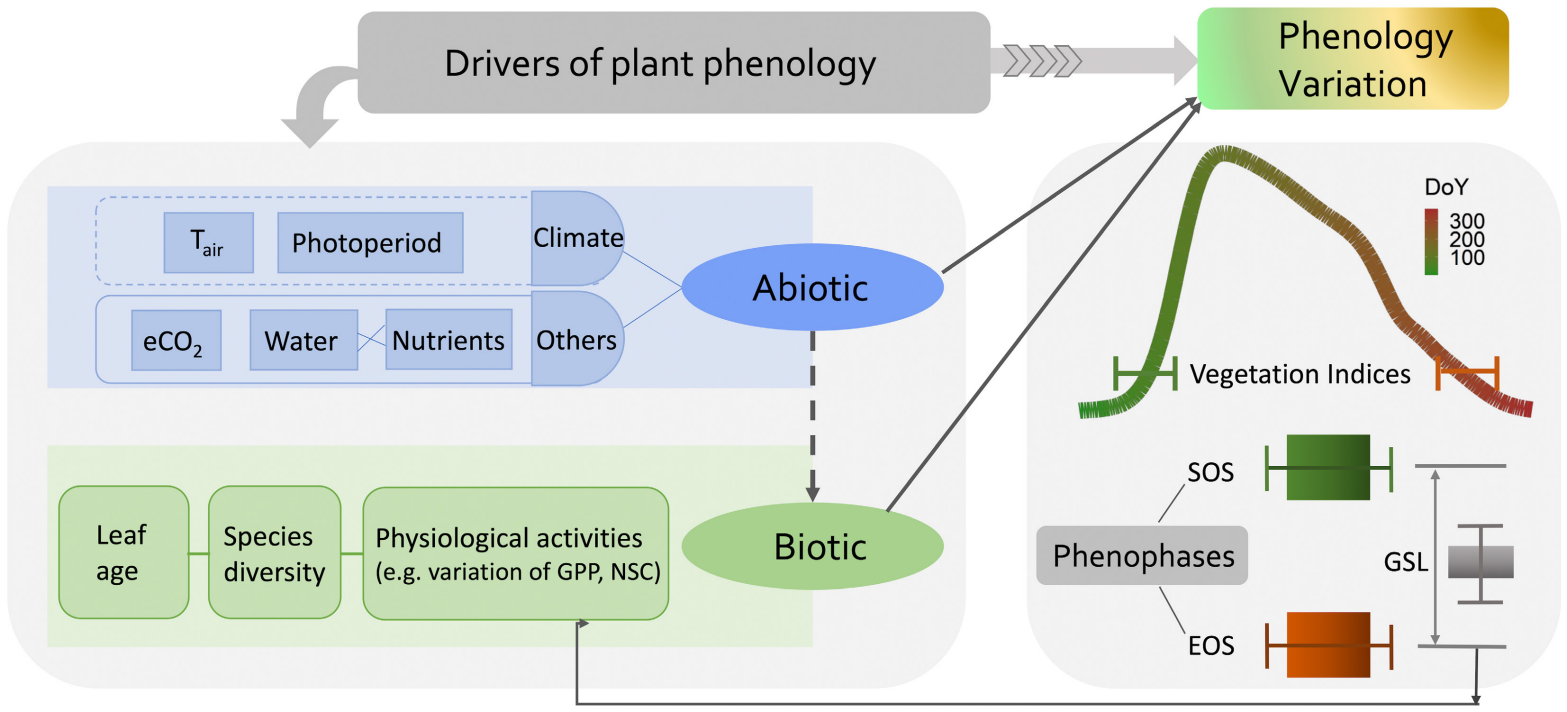
Overview of the biotic and abiotic drivers of plant phenology. The changes of critical phenophases such as start of the season (SOS), end of the season (EOS) are both influenced by abiotic and biotic factors, which further impact the growing season length (GSL) of vegetation growth and functioning. eCO_2_, elevated CO_2_; GPP, gross primary productivity; NSC, nonstructural carbon; *T*_air_, air temperature

Although much attention has been paid to reveal phenological patterns in space and time using satellite observations, identifying the underlying drivers and mechanisms of what we have observed using satellite observations is crucial to predict phenology changes in the future and accurately evaluate the phenology‐induced effects on ecosystem functioning (Piao et al., 2019). To date, much progress has been made to understand how climatic factors mediate the changes in phenology (Chamberlain & Wolkovich, 2021; Fu et al., 2015; Li, Liu, et al., 2021; Piao et al., 2015; Zohner et al., 2020). Key knowledge, however, remains highly lacking concerning the effects of drivers beyond climate (Figure 7). We are still limited to understanding how other environmental cues, regulating the leaf emergence and senescence, such as water, and nutrient availability (Luo et al., 2020; Piao et al., 2019), as well as elevated CO_2_ concentration (eCO_2_). Moreover, recent studies indicated biotic cues such as leaf age, species diversity, and physiological activities also have a strong impact on plant phenology (Chuine, 2010; Luo et al., 2022; Zani et al., 2020). For instance, increased photosynthesis was hypothesized to be the direct and determinant driver that advances the leaf senescence in the autumn and its importance of control on autumn phenology can differ in different growth periods. In this session, we mainly summarize the recent advances in studying the effects of factors beyond climate on phenology and discuss the possible directions to improve elucidating their effects on phenology with a combined remote sensing, experimentation, and modeling approach.

In addition to phenological cues such as temperature and day length, other environmental drivers such as water and nutrient availability, eCO_2_ can also influence the variation of phenology (Piao et al., 2019), especially for the sites that are limited by these factors (Chapin et al., 1990; Luo et al., 2020). For the drylands, water availability is a critical factor that can significantly influence the onset of SOS and EOS along with changes in soil water content (Luo et al., 2020; Reynolds et al., 2004). With the relief of limiting effects of water, nutrients' availability starts to play a more important role in regulating phenology (Guo et al., 2016; Lee et al., 2010; Luo et al., 2020). Increased soil water availability can help plants use more nutrients to synthesize organic matter and biomass during the growing season, which may enhance plants' resistance when facing stresses such as cold thus delaying senescence (Fu et al., 2019). At the same time, a threefold increase in anthropogenic nitrogen deposition with less obvious phosphorus deposition since the Industrial Revolution in 1860 has increased the ratio of nitrogen (N) to phosphorus (P). This leads to the so‐called N‐P imbalance and stoichiometry that are expected to have large impacts on ecosystem properties and dynamics of carbon and plant growth (Janssens et al., 2010; Nair et al., 2019). One landscape‐scale nutrient manipulation study in the Mediterranean tree‐grass ecosystem that involves the use of eddy‐covariance flux towers, phenocams, and satellite observations illustrated that nitrogen‐added treatment would accelerate the senescence rate and advance phenocam/satellite detected EOS compared with N:P balanced treatments (El‐Madany et al., 2021; Luo et al., 2020). This was attributed to the fact that soil water depleted more rapidly in the nitrogen‐added treatment during the dry‐down period due to enhanced leaf biomass production (Luo et al., 2020). Additionally, free air CO_2_ enrichment experiments can delay the timing of leaf senescence under elevated CO_2_, which might attribute to eCO_2_ improves the WUE (Ainsworth & Long, 2005; Norby, 2021; Reyes‐Fox et al., 2014) and ameliorates the soil water deficit (Fay et al., 2012).

From the recent report of the World Meteorological Organization (WMO), the global CO_2_ concentration continues to increase and already surpassed 415 ppm (WMO, 2022). Even though the amount of reactive nitrogen (Nr) has reached its peak worldwide, a large spatial variation of Nr deposition in different continents exists (Liu et al., 2022). Besides, we have a long road to clearly understanding the interactions between Nr and climate (Greaver et al., 2016). Due to the above reasons, there is large uncertainty to predict the spatiotemporal variation of plant phenology under future climate changes. Hence, multi‐factorial experiments, especially the interaction between different drivers are strongly needed in future work to elucidate the mechanisms and effects of environmental factors on phenology.

Apart from abiotic drivers, less studied biotic drivers also contribute to explaining the variation of phenology. Previous studies have demonstrated a positive intercorrelation between leaf onset and leaf senescence on species and ecosystem scales (Fu et al., 2014; Keenan & Richardson, 2015; Liu, Wu, et al., 2016). This phenomenon could be related to programmed cell death and relatively stable leaf longevity for specific species (Lim et al., 2007), as well as the consequence of the interaction of phenology and environmental drives, for example, earlier leaf‐out and expansion possibly result in advance of senescence by depleting limited water resources in the early stage of growing season (Luo et al., 2020; Wolf et al., 2016). Besides, leaf age and species diversity can also significantly influence the phenology and ecosystem functioning (Chuine, 2010; Wu et al., 2016). Studies on evergreen broadleaved trees reveal that leaf quantity as measured by LAI alone cannot explain the seasonality of photosynthesis capacity thus the same for the timing of important transition period (phenology) unless leaf age is considered (Wu et al., 2016, 2018). Likewise, for ecosystems with diverse species, phenology as an integrative indicator represents the variation of leaf development and greenness variation of different species, which would be affected if species composition is shifted (Chuine et al., 2010; Filippa et al., 2016) when facing environmental changes such as nutrients addition. However, the linkage between species changes and phenology variation, as well as their impact on ecosystem functioning (Cleland et al., 2007) have not been extensively studied.

A recent study proposed that enhancement of trees' photosynthesis in the growing season will advance autumn leaf senescence in temperate trees (Zani et al., 2020). If this is true, temperate and boreal ecosystems that are regarded as the important carbon sink might not continue to sink in the second half of the 21st century as the advance of leaf senescence and reduced growing season length in a CO_2_‐enriching atmosphere (Norby, 2021; Zani et al., 2020). Although strong controversy with respect to whether accumulated photosynthesis is the main regulator of autumn phenology or not (Lu & Keenan, 2022; Norby, 2021), the most updated studies on this topic have illustrated that phenology should not only be affected by climate but also regulated by physiological activities such as photosynthesis and variation/mobility of non‐structural carbon. Hence, more studies on biotic factors' effects as well as their relative importance compared with environmental factors on phenology are needed to be further investigated (Piao et al., 2019).

With rapid development in remote sensing techniques and their application, it is an opportunity and imperative to explore and bridge the underlying mechanisms of phenological variation at field scale and landscape scale by combining different approaches (i.e., experiments, observations from field measurements and remote sensing, and phenological modelling). Specifically, we can deepen the understanding of phenological changes through the following manners: (1) conducting manipulative experiments on the ecosystem scale (El‐Madany et al., 2018; Luo et al., 2018, 2020; Richardson, Hufkens, Milliman, Aubrecht, Chen, et al., 2018; Richardson, Hufkens, Milliman, Aubrecht, Furze, et al., 2018) that encompass different ecosystem components (e.g., overstorey and understorey, different plant species). By integrating data and knowledge from different sources, we can have an overview of the ecosystem phenological changes under the altered environment while elucidating the contributions from different ecosystem components and (2) fostering the development of the linkage between field and spatial observations. With the establishment of continental and global phenology and ecological monitoring networks, significant progress is made to connecting phenology information across spatial and temporal scales. This is particularly important for us to evaluate the consistency of phenology variation at different scales (Donnelly et al., 2022; Wang, Li, et al., 2022; Wang, Wu, et al., 2022), and further investigate the relationship between changes in environmental drivers, physiological activities, and variation of phenology (Lu & Keenan, 2022); (3) strengthening our ability to predict the spatiotemporal variation of phenology through modeling. With a wealth of data mentioned above becoming available, we not only gain the confidence to falsify the competing phenology hypotheses embedded into the global dynamic vegetation models (Hufkens et al., 2018; Richardson, 2019) but also can potentially add the overlooked processes in the models (Zani et al., 2020).

## 8 CONCLUDING REMARKS

In summary, after decades of development, LSP is becoming more and more mature in terms of data source, retrieval algorithm, and validation strategy. At the same time, the most important driving force of LSP is the demand from other fields for high‐quality and high‐resolution phenology information. This requires a close communication between the remote sensing community and scientists from other fields to foster mutual understanding. LSP is a research field that weaves together multiple disciplines, including remote sensing, climatology, agriculture, ecology, public health, and global change biology. It is perhaps worth to stress again the necessity of coupling technological advances to scientific questions as we elaborated in this review. We truly believe that only through a collaborative approach can we achieve both technically sound and scientifically meaningful global phenology monitoring goals.

## CONFLICT OF INTEREST

The authors declare that they have no conflict of interest.

## Data Availability

Data sharing is not applicable to this article as no data sets were generated or analyzed during the current study.

## References

[gcb16436-bib-0001] Ainsworth, E. A. , & Long, S. P. (2005). What have we learned from 15 years of free‐air CO_2_ enrichment (FACE)? A meta‐analytic review of the responses of photosynthesis, canopy properties and plant production to rising CO_2_ . New Phytologist, 165(2), 351–371. 10.1111/j.1469-8137.2004.01224.x 15720649

[gcb16436-bib-0002] Alemu, W. , & Henebry, G. (2016). Land surface phenology and seasonality using cool earthlight in croplands of eastern Africa and the linkages to crop production. Remote Sensing, 9(9), 914. 10.3390/rs9090914

[gcb16436-bib-0003] Atkinson, P. M. , Jeganathan, C. , Dash, J. , & Atzberger, C. (2012). Inter‐comparison of four models for smoothing satellite sensor time‐series data to estimate vegetation phenology. Remote Sensing of Environment, 123, 400–417. 10.1016/j.rse.2012.04.001

[gcb16436-bib-0004] Baumann, M. , Ozdogan, M. , Richardson, A. D. , & Radeloff, V. C. (2017). Phenology from Landsat when data is scarce: Using MODIS and Dynamic Time‐Warping to combine multi‐year Landsat imagery to derive annual phenology curves. International Journal of Applied Earth Observation and Geoinformation, 54, 72–83. 10.1016/j.jag.2016.09.005

[gcb16436-bib-0005] Beer, C. , Ciais, P. , Reichstein, M. , Baldocchi, D. , Law, B. E. , Papale, D. , Soussana, J. F. , Ammann, C. , Buchmann, N. , Frank, D. , Gianelle, D. , Janssens, I. A. , Knohl, A. , Köstner, B. , Moors, E. , Roupsard, O. , Verbeeck, H. , Vesala, T. , Williams, C. A. , & Wohlfahrt, G. (2009). Temporal and among‐site variability of inherent water use efficiency at the ecosystem level. Global Biogeochemical Cycles, 23(2), GB2018. 10.1029/2008GB003233

[gcb16436-bib-0006] Berra, E. F. , & Gaulton, R. (2021). Remote sensing of temperate and boreal forest phenology: A review of progress, challenges and opportunities in the intercomparison of in‐situ and satellite phenological metrics. Forest Ecology and Management, 480, 118663. 10.1016/j.foreco.2020.118663

[gcb16436-bib-0007] Bison, N. N. , Partelli‐Feltrin, R. , & Michaletz, S. T. (2022). Trait phenology and fire seasonality co‐drive seasonal variation in fire effects on tree crowns. New Phytologist, 234(5), 1654–1663. 10.1111/nph.18047 35181920

[gcb16436-bib-0008] Bogawski, P. , Borycka, K. , Grewling, L. , & Kasprzyk, I. (2019). Detecting distant sources of airborne pollen for Poland: Integrating back‐trajectory and dispersion modelling with a satellite‐based phenology. Science of the Total Environment, 689, 109–125. 10.1016/j.scitotenv.2019.06.348 31271980

[gcb16436-bib-0009] Boukabara, S.‐A. , Krasnopolsky, V. , Penny, S. G. , Stewart, J. Q. , McGovern, A. , Hall, D. , Ten Hoeve, J. E. , Hickey, J. , Allen Huang, H.‐L. , Williams, J. K. , Ide, K. , Tissot, P. , Haupt, S. E. , Casey, K. S. , Oza, N. , Geer, A. J. , Maddy, E. S. , & Hoffman, R. N. (2021). Outlook for exploiting artificial intelligence in the earth and environmental sciences. Bulletin of the American Meteorological Society, 102(5), 1016–1032. 10.1175/BAMS-D-20-0031.1

[gcb16436-bib-0010] Breckheimer, I. K. , Theobald, E. J. , Cristea, N. C. , Wilson, A. K. , Lundquist, J. D. , Rochefort, R. M. , & HilleRisLambers, J. (2019). Crowd‐sourced data reveal social‐ecological mismatches in phenology driven by climate. Frontiers in Ecology and the Environment, 18(2), 76–82. 10.1002/fee.2142

[gcb16436-bib-0011] Brown, L. A. , Dash, J. , Ogutu, B. O. , & Richardson, A. D. (2017). On the relationship between continuous measures of canopy greenness derived using near‐surface remote sensing and satellite‐derived vegetation products. Agricultural and Forest Meteorology, 247, 280–292. 10.1016/j.agrformet.2017.08.012

[gcb16436-bib-0012] Brown, T. B. , Hultine, K. R. , Steltzer, H. , Denny, E. G. , Denslow, M. W. , Granados, J. , Henderson, S. , Moore, D. , Nagai, S. , SanClements, M. , Sánchez‐Azofeifa, A. , Sonnentag, O. , Tazik, D. , & Richardson, A. D. (2016). Using PhenoCams to monitor our changing Earth: Toward a Global PhenoCam Network. Frontiers in Ecology and the Environment, 14(2), 84–93. 10.1002/fee.1222

[gcb16436-bib-0013] Browning, D. M. , Russell, E. S. , Ponce‐Campos, G. E. , Kaplan, N. , Richardson, A. D. , Seyednasrollah, B. , Spiegal, S. , Saliendra, N. , Alfieri, J. G. , Baker, J. , Bernacchi, C. , Bestelmeyer, B. T. , Bosch, D. , Boughton, E. H. , Boughton, R. K. , Gerald Flerchinger, P. C. , Gomez‐Casanovas, N. , Goslee, S. , Haddad, N. M. , … Taylor, S. D. (2021). Monitoring agroecosystem productivity and phenology at a national scale: A metric assessment framework. Ecological Indicators, 131, 108147. 10.1016/j.ecolind.2021.108147

[gcb16436-bib-0014] Buermann, W. , Bikash, P. R. , Jung, M. , Burn, D. H. , & Reichstein, M. (2013). Earlier springs decrease peak summer productivity in North American boreal forests. Environmental Research Letters, 8(2), 024027. 10.1088/1748-9326/8/2/024027

[gcb16436-bib-0015] Cai, Z. , Jönsson, P. , Jin, H. , & Eklundh, L. (2017). Performance of smoothing methods for reconstructing NDVI time‐series and estimating vegetation phenology from MODIS data. Remote Sensing, 9(12), 1271. 10.3390/rs9121271

[gcb16436-bib-0016] Campbell, B. C. , Kouba, J. , Timbrell, V. , Noor, M. , Massel, K. , Gilding, E. , Angel, N. , Kemish, B. , Hugenholtz, P. , Godwin, I. , & Davies, J. (2020). Tracking seasonal changes in diversity of pollen allergen exposure: Targeted metabarcoding of a subtropical aerobiome. Science of the Total Environment, 747, 1411891. 10.1016/j.scitotenv.2020.141189 32799020

[gcb16436-bib-0017] Caparros‐Santiago, J. A. , Rodriguez‐Galiano, V. , & Dash, J. (2021). Land surface phenology as indicator of global terrestrial ecosystem dynamics: A systematic review. ISPRS Journal of Photogrammetry and Remote Sensing, 171, 330–347. 10.1016/j.isprsjprs.2020.11.019

[gcb16436-bib-0018] Chamberlain, C. J. , & Wolkovich, E. M. (2021). Late spring freezes coupled with warming winters alter temperate tree phenology and growth. New Phytologist, 231(3), 987–995. 10.1111/nph.17416 33932291

[gcb16436-bib-0019] Chapin, F. S. , Schulze, E.‐D. , & Mooney, H. A. (1990). The ecology and economics of storage in plants. Annual Review of Ecology and Systematics, 21, 423–447. 10.1146/annurev.es.21.110190.002231

[gcb16436-bib-0020] Chen, J. , Jönsson, P. , Tamura, M. , Gu, Z. , Matsushita, B. , & Eklundh, L. (2004). A simple method for reconstructing a high‐quality NDVI time‐series data set based on the Savitzky–Golay filter. Remote Sensing of Environment, 91(3–4), 332–344. 10.1016/j.rse.2004.03.014

[gcb16436-bib-0021] Chen, X. , Wang, D. , Chen, J. , Wang, C. , & Shen, M. (2018). The mixed pixel effect in land surface phenology: A simulation study. Remote Sensing of Environment, 211, 338–344. 10.1016/j.rse.2018.04.030

[gcb16436-bib-0022] Chuine, I. (2010). Why does phenology drive species distribution? Philosophical Transactions of the Royal Society B: Biological Sciences, 365(1555), 3149–3160. 10.1098/rstb.2010.0142 PMC2981946 20819809

[gcb16436-bib-0023] Claverie, M. , Ju, J. , Masek, J. G. , Dungan, J. L. , Vermote, E. F. , Roger, J.‐C. , Skakun, S. V. , & Justice, C. (2018). The Harmonized Landsat and Sentinel‐2 surface reflectance product. Remote Sensing of Environment, 219, 145–161. 10.1016/j.rse.2018.09.002

[gcb16436-bib-0024] Cleland, E. E. , Chuine, I. , Menzel, A. , Mooney, H. A. , & Schwartz, M. D. (2007). Shifting plant phenology in response to global change. Trends in Ecology & Evolution, 22(7), 357–365. 10.1016/j.tree.2007.04.003 17478009

[gcb16436-bib-0025] Cong, N. , Piao, S. , Chen, A. , Wang, X. , Lin, X. , Chen, S. , Han, S. , Zhou, G. , & Zhang, X. (2012). Spring vegetation green‐up date in China inferred from SPOT NDVI data: A multiple model analysis. Agricultural and Forest Meteorology, 165, 104–113. 10.1016/j.agrformet.2012.06.009

[gcb16436-bib-0026] Cope, M. , Mikhailova, E. , Post, C. , Schlautman, M. , & McMillan, P. (2017). Developing an integrated cloud‐based spatial‐temporal system for monitoring phenology. Ecological Informatics, 39, 123–129. 10.1016/j.ecoinf.2017.04.007

[gcb16436-bib-0027] Correia, D. L. P. , Bouachir, W. , Gervais, D. , Pureswaran, D. , Kneeshaw, D. D. , & De Grandpre, L. (2020). Leveraging artificial intelligence for large‐scale plant phenology studies from noisy time‐lapse images. IEEE Access, 8, 13151–13160. 10.1109/ACCESS.2020.2965462

[gcb16436-bib-0028] Dai, J. , Wang, H. , & Ge, Q. (2013). The spatial pattern of leaf phenology and its response to climate change in China. International Journal of Biometeorology, 58(4), 521–528. 10.1007/s00484-013-0679-2 23732443

[gcb16436-bib-0029] d'Andrimont, R. , Yordanov, M. , Martinez‐Sanchez, L. , & van der Velde, M. (2022). Monitoring crop phenology with street‐level imagery using computer vision. Computers and Electronics in Agriculture, 196, 106866. 10.1016/j.compag.2022.106866

[gcb16436-bib-0030] Davies, J. M. , Beggs, P. J. , Medek, D. E. , Newnham, R. M. , Erbas, B. , Thibaudon, M. , Katelaris, C. H. , Haberle, S. G. , Newbigin, E. J. , & Huete, A. R. (2015). Trans‐disciplinary research in synthesis of grass pollen aerobiology and its importance for respiratory health in Australasia. Science of the Total Environment, 534, 85–96. 10.1016/j.scitotenv.2015.04.001 25891684

[gcb16436-bib-0031] Davies, J. M. , Berman, D. , Beggs, P. , Dario Ramon, G. , Jonny Peter, M. D. , Katelaria, C. , & Ziska, L. (2021). Global climate change and pollen aeroallergens: A southern hemisphere perspective. Immunology and Allergy Clinics, 41(1), 1–16. 10.1016/j.iac.2020.09.002 33228867

[gcb16436-bib-0032] de Beurs, K. M. , & Henebry, G. M. (2008). War, drought, and phenology: Changes in the land surface phenology of Afghanistan since 1982. Journal of Land Use Science, 3(2–3), 95–111. 10.1080/17474230701786109

[gcb16436-bib-0033] Devadas, R. , Huete, A. R. , Vicendese, D. , Erbas, B. , Beggs, P. J. , Medek, D. , Haberle, S. G. , Newnham, R. M. , Johnston, F. H. , Jaggard, A. K. , Campbell, B. , Burton, P. K. , Katelaris, C. H. , Newbigin, E. , Thibaudon, M. , & Davies, J. M. (2018). Dynamic ecological observations from satellites inform aerobiology of allergenic grass pollen. Science of the Total Environment, 633, 441–451. 10.1016/j.scitotenv.2018.03.191 29579655

[gcb16436-bib-0034] Dixon, D. J. , Callow, N. , Duncan, M. J. , Setterfield, S. A. , & Pauli, N. (2021). Satellite prediction of forest flowering phenology. Remote Sensing of Environment, 255, 112197. 10.1016/j.rse.2020.112197

[gcb16436-bib-0035] Donnelly, A. , Yu, R. , Jones, K. , Belitz, M. , Li, B. , Duffy, K. , Zhang, X. , Wang, J. , Seyednasrollah, B. , Gerst, K. L. , Li, D. , Kaddoura, Y. , Zhu, K. , Morisette, J. , Ramey, C. , & Smith, K. (2022). Exploring discrepancies between in situ phenology and remotely derived phenometrics at NEON sites. Ecosphere, 13(1), e3912. 10.1002/ecs2.3912

[gcb16436-bib-0036] Dye, D. G. (2002). Variability and trends in the annual snow‐cover cycle in Northern Hemisphere land areas, 1972–2000. Hydrological Processes, 16(15), 3065–3077. 10.1002/hyp.1089

[gcb16436-bib-0037] El‐Madany, T. S. , Reichstein, M. , Carrara, A. , Martín, M. P. , Moreno, G. , Gonzalez‐Cascon, R. , Peñuelas, J. , Ellsworth, D. S. , Burchard‐Levine, V. , Hammer, T. W. , Knauer, J. , Kolle, O. , Luo, Y. , Pacheco‐Labrador, J. , Nelson, J. A. , Perez‐Priego, O. , Rolo, V. , Wutzler, T. , & Migliavacca, M. (2021). How nitrogen and phosphorus availability change water use efficiency in a mediterranean savanna ecosystem. Journal of Geophysical Research: Biogeosciences, 126(5), e2020JG006005. 10.1029/2020JG006005

[gcb16436-bib-0038] El‐Madany, T. S. , Reichstein, M. , Perez‐Priego, O. , Carrara, A. , Moreno, G. , Pilar Martín, M. , Pacheco‐Labrador, J. , Wohlfahrt, G. , Nieto, H. , Weber, U. , Kolle, O. , Luo, Y. , Carvalhais, N. , & Migliavacca, M. (2018). Drivers of spatio‐temporal variability of carbon dioxide and energy fluxes in a Mediterranean savanna ecosystem. Agricultural and Forest Meteorology, 262, 258–278. 10.1016/j.agrformet.2018.07.010

[gcb16436-bib-0039] Elmore, A. , Stylinski, C. , & Pradhan, K. (2016). Synergistic use of citizen science and remote sensing for continental‐scale measurements of forest tree phenology. Remote Sensing, 8(6), 502. 10.3390/rs8060502

[gcb16436-bib-0040] Emmerson, K. M. , Silver, J. D. , Newbigin, E. , Lampugnani, E. R. , Suphioglu, C. , Wain, A. , & Ebert, E. (2019). Development and evaluation of pollen source methodologies for the Victorian Grass Pollen Emissions Module VGPEM1.0. Geoscientific Model Development, 12, 2195–2214. 10.5194/gmd-12-2195-2019

[gcb16436-bib-0041] Fay, P. A. , Jin, V. L. , Way, D. A. , Potter, K. N. , Gill, R. A. , Jackson, R. B. , & Wayne Polley, H. (2012). Soil‐mediated effects of subambient to increased carbon dioxide on grassland productivity. Nature Climate Change, 2(10), 742–746. 10.1038/nclimate1573

[gcb16436-bib-0042] Filippa, G. , Cremonese, E. , Migliavacca, M. , Galvagno, M. , Forkel, M. , Wingate, L. , Tomelleri, E. , Morra di Cella, U. , & Richardson, A. D. (2016). Phenopix: A R package for image‐based vegetation phenology. Agricultural and Forest Meteorology, 220, 141–150. 10.1016/J.AGRFORMET.2016.01.006

[gcb16436-bib-0043] Friedl, M. A. , Gray, J. M. , Melaas, E. K. , Richardson, A. D. , Hufkens, K. , Keenan, T. F. , Bailey, A. , & O'Keefe, J. (2014). A tale of two springs: Using recent climate anomalies to characterize the sensitivity of temperate forest phenology to climate change. Environmental Research Letters, 9(5), 054006. 10.1088/1748-9326/9/5/054006

[gcb16436-bib-0044] Fu, Y. , Li, X. , Zhou, X. , Geng, X. , Guo, Y. , & Zhang, Y. (2020). Progress in plant phenology modeling under global climate change. Science China Earth Sciences, 63(9), 1237–1247. 10.1007/s11430-019-9622-2

[gcb16436-bib-0045] Fu, Y. H. , Piao, S. , Delpierre, N. , Hao, F. , Hanninen, H. , Geng, X. , Penuelas, J. , Zhang, X. , Janssens, I. A. , & Campioli, M. (2019). Nutrient availability alters the correlation between spring leaf‐out and autumn leaf senescence dates. Tree Physiology, 39(8), 1277–1284. 10.1093/treephys/tpz041 30989235

[gcb16436-bib-0046] Fu, Y. H. , Piao, S. , Op de Beeck, M. , Cong, N. , Zhao, H. , Zhang, Y. , Menzel, A. , & Janssens, I. A. (2014). Recent spring phenology shifts in western Central Europe based on multiscale observations. Global Ecology and Biogeography, 23(11), 1255–1263. 10.1111/geb.12210

[gcb16436-bib-0047] Fu, Y. H. , Piao, S. , Vitasse, Y. , Zhao, H. , De Boeck, H. J. , Liu, Q. , Yang, H. , Weber, U. , Hanninen, H. , & Janssens, I. A. (2015). Increased heat requirement for leaf flushing in temperate woody species over 1980–2012: Effects of chilling, precipitation and insolation. Global Change Biology, 21(7), 2687–2697. 10.1111/gcb.12863 25580596

[gcb16436-bib-0048] Gao, F. , & Zhang, X. (2021). Mapping crop phenology in near real‐time using satellite remote sensing: Challenges and opportunities. Journal of Remote Sensing, 2021, 1–14 10.34133/2021/8379391.

[gcb16436-bib-0049] García‐Mozo, H. , Galan, C. , Belmonte, J. , Bermejo, D. , Candau, P. , Diazdelaguardia, C. , Elvira, B. , Gutierrez, M. , Jato, V. , & Silva, I. (2009). Predicting the start and peak dates of the Poaceae pollen season in Spain using process‐based models. Agricultural and Forest Meteorology, 149(2), 256–262. 10.1016/j.agrformet.2008.08.013

[gcb16436-bib-0050] Ge, Q. , Wang, H. , & Dai, J. (2013). Shifts in spring phenophases, frost events and frost risk for woody plants in temperate China. Climate Research, 57, 249–258. 10.3354/cr01182

[gcb16436-bib-0051] Gervais, N. , Buyantuev, A. , & Gao, F. (2017). Modeling the effects of the urban built‐up environment on plant phenology using fused satellite data. Remote Sensing, 9(1), 99. 10.3390/rs9010099

[gcb16436-bib-0052] Gray, J. , Friedl, M. , Frolking, S. , Ramankutty, N. , Nelson, A. , & Gumma, M. K. (2014). Mapping Asian cropping intensity with MODIS. IEEE Journal of Selected Topics in Applied Earth Observations and Remote Sensing, 7(8), 3373–3379. 10.1109/JSTARS.2014.2344630

[gcb16436-bib-0053] Gray, J. M. , Frolking, S. , Kort, E. A. , Ray, D. K. , Kucharik, C. J. , Ramankutty, N. , & Friedl, M. A. (2014). Direct human influence on atmospheric CO_2_ seasonality from increased cropland productivity. Nature, 515(7527), 398–401. 10.1038/nature13957 25409830

[gcb16436-bib-0054] Greaver, T. , Clark, C. , Compton, J. , Vallano, D. , Talhelm, A. , Weaver, C. , Band, L. , Baron, J. , Davidson, E. , Tague, C. , Felker‐Quinn, E. , Lynch, J. , Herrick, J. , Liu, L. , Goodale, C. , Novak, K. , & Haeuber, R. (2016). Key ecological responses to nitrogen are altered by climate change. Nature Climate Change, 6(9), 836–843. 10.1038/nclimate3088

[gcb16436-bib-0055] Guo, Q. , Hu, Z. M. , Li, S. G. , Yu, G. R. , Sun, X. M. , Li, L. H. , Liang, N. S. , & Bai, W. M. (2016). Exogenous N addition enhances the responses of gross primary productivity to individual precipitation events in a temperate grassland. Scientific Reports, 6, 26901. 10.1038/srep26901 27264386 PMC4893632

[gcb16436-bib-0056] Han, G. , & Xu, J. (2013). Land surface phenology and land surface temperature changes along an urban‐rural gradient in Yangtze River Delta, China. Environment Management, 52(1), 234–249. 10.1007/s00267-013-0097-6 23740439

[gcb16436-bib-0057] Hänninen, H. (2006). Climate warming and the risk of frost damage to boreal forest trees: Identification of critical ecophysiological traits. Tree Physiology, 26(7), 889–898. 10.1093/treephys/26.7.889 16585034

[gcb16436-bib-0058] Hassan, M. A. , Yang, M. , Rasheed, A. , Yang, G. , Reynolds, M. , Xia, X. , Xiao, Y. , & He, Z. (2019). A rapid monitoring of NDVI across the wheat growth cycle for grain yield prediction using a multi‐spectral UAV platform. Plant Science, 282, 95–103. 10.1016/j.plantsci.2018.10.022 31003615

[gcb16436-bib-0059] Huete, A. , Tran, N. N. , Nguyen, H. , Xie, Q. , & Katelaris, C. (2019). Forecasting pollen aerobiology with NODIS EVI, land cover, and phenology using machine learning tools. *IGARSS 2019‐2019 IEEE International Geoscience and Remote Sensing Symposium*. *IEEE*, 5429–5432. 10.1109/IGARSS.2019.8898796

[gcb16436-bib-0060] Hufkens, K. , Basler, D. , Milliman, T. , Melaas, E. K. , & Richardson, A. D. (2018). An integrated phenology modelling framework in R. Methods in Ecology and Evolution, 9(5), 1276–1285. 10.1111/2041-210X.12970

[gcb16436-bib-0061] Hufkens, K. , Melaas, E. K. , Mann, M. L. , Foster, T. , Ceballos, F. , Robles, M. , & Kramer, B. (2019). Monitoring crop phenology using a smartphone based near‐surface remote sensing approach. Agricultural and Forest Meteorology, 265, 327–337. 10.1016/j.agrformet.2018.11.002

[gcb16436-bib-0062] Inouye, D. W. (2022). Climate change and phenology. Wiley Interdisciplinary Reviews: Climate Change, 13, e764. 10.1002/wcc.764

[gcb16436-bib-0063] Irrgang, C. , Boers, N. , Sonnewald, M. , Barnes, E. A. , Kadow, C. , Joanna, S. , & Saynisch‐Wagner, J. (2021). Towards neural Earth system modelling by integrating artificial intelligence in Earth system science. Nature Machine Intelligence, 3(8), 667–674. 10.1038/s42256-021-00374-3

[gcb16436-bib-0064] Janssens, I. A. , Dieleman, W. , Luyssaert, S. , Subke, J. A. , Reichstein, M. , Ceulemans, R. , Ciais, P. , Dolman, A. J. , Grace, J. , Matteucci, G. , Papale, D. , Piao, S. L. , Schulze, E. D. , Tang, J. , & Law, B. E. (2010). Reduction of forest soil respiration in response to nitrogen deposition. Nature Geoscience, 3(5), 315–322. 10.1038/ngeo844

[gcb16436-bib-0065] Jeganathan, C. , Dash, J. , & Atkinson, P. M. (2014). Remotely sensed trends in the phenology of northern high latitude terrestrial vegetation, controlling for land cover change and vegetation type. Remote Sensing of Environment, 143, 154–170. 10.1016/j.rse.2013.11.020

[gcb16436-bib-0066] Jin, H. , Jönsson, A. M. , Bolmgren, K. , Langvall, O. , & Eklundh, L. (2017). Disentangling remotely‐sensed plant phenology and snow seasonality at northern Europe using MODIS and the plant phenology index. Remote Sensing of Environment, 198, 203–212. 10.1016/j.rse.2017.06.015

[gcb16436-bib-0067] Jin, H. , Jonsson, A. M. , Olsson, C. , Lindstrom, J. , Jonsson, P. , & Eklundh, L. (2019). New satellite‐based estimates show significant trends in spring phenology and complex sensitivities to temperature and precipitation at northern European latitudes. International Journal of Biometeorology, 63(6), 763–775. 10.1007/s00484-019-01690-5 30805728

[gcb16436-bib-0068] Jin, J. , Wang, Y. , Zhang, Z. , Magliulo, V. , Jiang, H. , & Cheng, M. (2017). Phenology plays an important role in the regulation of terrestrial ecosystem water‐use efficiency in the northern hemisphere. Remote Sensing, 9(7), 664. 10.3390/rs9070664

[gcb16436-bib-0069] Jin, J. , Zhan, W. , Wang, Y. , Gu, B. , Wang, W. , Jiang, H. , Lu, X. , & Zhang, X. (2017). Water use efficiency in response to interannual variations in flux‐based photosynthetic onset in temperate deciduous broadleaf forests. Ecological Indicators, 79, 122–127. 10.1016/j.ecolind.2017.04.006

[gcb16436-bib-0070] Ju, J. , & Roy, D. P. (2008). The availability of cloud‐free Landsat ETM+ data over the conterminous United States and globally. Remote Sensing of Environment, 112(3), 1196–1211. 10.1016/j.rse.2007.08.011

[gcb16436-bib-0071] Keenan, T. F. , Gray, J. , Friedl, M. A. , Toomey, M. , Bohrer, G. , Hollinger, D. Y. , Munger, J. W. , O'Keefe, J. , Schmid, H. P. , Wing, I. S. , Yang, B. , & Richardson, A. D. (2014). Net carbon uptake has increased through warming‐induced changes in temperate forest phenology. Nature Climate Change, 4(7), 598–604. 10.1038/nclimate2253

[gcb16436-bib-0072] Keenan, T. F. , Hollinger, D. Y. , Bohrer, G. , Dragoni, D. , Munger, J. W. , Schmid, H. P. , & Richardson, A. D. (2013). Increase in forest water‐use efficiency as atmospheric carbon dioxide concentrations rise. Nature, 499(7458), 324–327. 10.1038/nature12291 23842499

[gcb16436-bib-0073] Keenan, T. F. , & Richardson, A. D. (2015). The timing of autumn senescence is affected by the timing of spring phenology: Implications for predictive models. Global Change Biology, 21(7), 2634–2641. 10.1111/gcb.12890 25662890

[gcb16436-bib-0074] Khare, S. , Deslauriers, A. , Morin, H. , Latifi, H. , & Rossi, S. (2021). Comparing time‐lapse PhenoCams with satellite observations across the boreal forest of Quebec, Canada. Remote Sensing, 14(1), 100. 10.3390/rs14010100

[gcb16436-bib-0075] Khwarahm, N. R. , Dash, J. , Skjoth, C. A. , Newnham, R. M. , Adams‐Groom, B. , Head, K. , Caulton, E. , & Atkinson, P. M. (2017). Mapping the birch and grass pollen seasons in the UK using satellite sensor time‐series. Science of the Total Environment, 578, 586–600. 10.1016/j.scitotenv.2016.11.004 27856057

[gcb16436-bib-0076] Kim, J. , Kim, Y. , Zona, D. , Oechel, W. , Park, S. J. , Lee, B. Y. , Yi, Y. , Erb, A. , & Schaaf, C. L. (2021). Carbon response of tundra ecosystems to advancing greenup and snowmelt in Alaska. Nature Communications, 12, 6879. 10.1038/s41467-021-26876-7 PMC8617207 34824215

[gcb16436-bib-0077] Kljun, N. , Black, T. A. , Griffis, T. J. , Barr, A. G. , Gaumont‐Guay, D. , Morgenstern, K. , McCaughey, J. H. , & Nesic, Z. (2006). Response of net ecosystem productivity of three boreal forest stands to drought. Ecosystems, 9(7), 1128–1144. 10.1007/s10021-007-9088-x

[gcb16436-bib-0078] Klosterman, S. T. , Hufkens, K. , Gray, J. M. , Melaas, E. , Sonnentag, O. , Lavine, I. , Mitchell, L. , Norman, R. , Friedl, M. A. , & Richardson, A. D. (2014). Evaluating remote sensing of deciduous forest phenology at multiple spatial scales using PhenoCam imagery. Biogeosciences, 11(16), 4305–4320. 10.5194/bg-11-4305-2014

[gcb16436-bib-0079] Lee, M. , Manning, P. , Rist, J. , Power, S. A. , & Marsh, C. (2010). A global comparison of grassland biomass responses to CO_2_ and nitrogen enrichment. Philosophical Transactions of the Royal Society B: Biological Sciences, 365(1549), 2047–2056. 10.1098/rstb.2010.0028 PMC2880131 20513713

[gcb16436-bib-0080] Leuzinger, S. , Zotz, G. , Asshoff, R. , & Korner, C. (2005). Responses of deciduous forest trees to severe drought in Central Europe. Tree Physiology, 25, 641–650. 10.1093/treephys/25.6.641 15805084

[gcb16436-bib-0081] Li, P. , Liu, Z. , Zhou, X. , Xie, B. , Li, Z. , Luo, Y. , Zhu, Q. , & Peng, C. (2021). Combined control of multiple extreme climate stressors on autumn vegetation phenology on the Tibetan Plateau under past and future climate change. Agricultural and Forest Meteorology, 308–309, 108571. 10.1016/j.agrformet.2021.108571

[gcb16436-bib-0082] Li, Q. , Shen, M. , Chen, X. , Wang, C. , Chen, J. , Cao, X. , & Cui, X. (2021). Optimal color composition method for generating high‐quality daily photographic time series from PhenoCam. IEEE Journal of Selected Topics in Applied Earth Observations and Remote Sensing, 14, 6179–6193. 10.1109/JSTARS.2021.3087814

[gcb16436-bib-0083] Li, X. , Zhou, Y. , Asrar, G. R. , Mao, J. , Li, X. , & Li, W. (2016). Response of vegetation phenology to urbanization in the conterminous United States. Global Change Biology, 23(7), 2818–2830. 10.1111/gcb.13562 27988975

[gcb16436-bib-0084] Li, X. , Zhou, Y. , Meng, L. , Asrar, G. , Sapkota, A. , & Coates, F. (2019). Characterizing the relationship between satellite phenology and pollen season: A case study of birch. Remote Sensing of Environment, 222, 267–274. 10.1016/j.rse.2018.12.036

[gcb16436-bib-0085] Lim, P. O. , Kim, H. J. , & Nam, H. G. (2007). Leaf senescence. Annual Review of Plant Biology, 58, 115–136. 10.1146/annurev.arplant.57.032905.105316 17177638

[gcb16436-bib-0086] Liu, L. , Cao, R. , Shen, M. , Chen, J. , Wang, J. , & Zhang, X. (2019). How does scale effect influence spring vegetation phenology estimated from satellite‐derived vegetation indexes? Remote Sensing, 11(18), 2137. 10.3390/rs11182137

[gcb16436-bib-0087] Liu, L. , Xu, W. , Lu, X. , Zhong, B. , Guo, Y. , Lu, X. , Zhao, Y. , He, W. , Wang, S. , Zhang, X. , Liu, X. , & Vitousek, P. (2022). Exploring global changes in agricultural ammonia emissions and their contribution to nitrogen deposition since 1980. Proceedings of the National Academy of Sciences of the United States of America, 119(14), e2121998119. 10.1073/pnas.2121998119 35344440 PMC9169101

[gcb16436-bib-0088] Liu, N. , Garcia, M. , Singh, A. , Clare, J. D. J. , Stenglein, J. L. , Zuckerberg, B. , Kruger, E. L. , & Townsend, P. A. (2021). Trail camera networks provide insights into satellite‐derived phenology for ecological studies. International Journal of Applied Earth Observation and Geoinformation, 97, 102291. 10.1016/j.jag.2020.102291

[gcb16436-bib-0089] Liu, Q. , Fu, Y. H. , Zhu, Z. , Liu, Y. , Liu, Z. , Huang, M. , Janssens, I. A. , & Piao, S. (2016). Delayed autumn phenology in the Northern Hemisphere is related to change in both climate and spring phenology. Global Change Biology, 22(11), 3702–3711. 10.1111/gcb.13311 27061925

[gcb16436-bib-0090] Liu, Y. , Hill, M. J. , Zhang, X. , Wang, Z. , Richardson, A. D. , Hufkens, K. , Filippa, G. , Baldocchi, D. D. , Ma, S. , Verfaillie, J. , & Schaaf, C. B. (2017). Using data from Landsat, MODIS, VIIRS and PhenoCams to monitor the phenology of California oak/grass savanna and open grassland across spatial scales. Agricultural and Forest Meteorology, 237–238, 311–325. 10.1016/j.agrformet.2017.02.026

[gcb16436-bib-0091] Liu, Y. , & Wu, C. (2020). Understanding the role of phenology and summer physiology in controlling net ecosystem production: a multiscale comparison of satellite, PhenoCam and eddy covariance data. Environmental Research Letters, 15(10), 104086. 10.1088/1748-9326/abb32f

[gcb16436-bib-0092] Liu, Y. , Wu, C. , Peng, D. , Xu, S. , Gonsamo, A. , Jassal, R. S. , Altaf Arain, M. , Lu, L. , Fang, B. , & Chen, J. M. (2016). Improved modeling of land surface phenology using MODIS land surface reflectance and temperature at evergreen needleleaf forests of central North America. Remote Sensing of Environment, 176, 152–162. 10.1016/j.rse.2016.01.021

[gcb16436-bib-0093] Lobell, D. B. , Burke, M. B. , Tebaldi, C. , Mastrandrea, M. D. , Falcon, W. P. , & Naylor, R. L. (2008). Prioritizing climate change adaptation needs for food security in 2030. Science, 319(5863), 607–610. 10.1126/science.1152339 18239122

[gcb16436-bib-0094] Lu, J. , He, T. , Song, D.‐X. , & Wang, C.‐Q. (2022). Land surface phenology retrieval through spectral and angular harmonization of Landsat‐8, Sentinel‐2 and Gaofen‐1 data. Remote Sensing, 14(5), 1296. 10.3390/rs14051296

[gcb16436-bib-0095] Lu, X. , & Keenan, T. F. (2022). No evidence for a negative effect of growing season photosynthesis on leaf senescence timing. Global Change Biology, 28(9), 3083–3093. 10.1111/gcb.16104 35174579

[gcb16436-bib-0096] Luo, Y. , El‐Madany, T. , Ma, X. , Nair, R. , Jung, M. , Weber, U. , Filippa, G. , Bucher, S. F. , Moreno, G. , Cremonese, E. , Carrara, A. , Gonzalez‐Cascon, R. , Caceres Escudero, Y. , Galvagno, M. , Pacheco‐Labrador, J. , Martin, M. P. , Perez‐Priego, O. , Reichstein, M. , Richardson, A. D. , … Migliavacca, M. (2020). Nutrients and water availability constrain the seasonality of vegetation activity in a Mediterranean ecosystem. Global Change Biology, 26(8), 4379–4400. 10.1111/gcb.15138 32348631

[gcb16436-bib-0097] Luo, Y. , El‐Madany, T. S. , Filippa, G. , Ma, X. , Ahrens, B. , Carrara, A. , Gonzalez‐Cascon, R. , Cremonese, E. , Galvagno, M. , Hammer, T. , Pacheco‐Labrador, J. , Pilar Martin, M. , Moreno, G. , Perez‐Priego, O. , Reichstein, M. , Richardson, A. , Romermann, C. , & Migliavacca, M. (2018). Using near‐infrared‐enabled digital repeat photography to track structural and physiological phenology in mediterranean tree–grass ecosystems. Remote Sensing, 10(8), 1293. 10.3390/rs10081293

[gcb16436-bib-0098] Luo, Y. , Pacheco‐Labrador, J. , Richardson, A. D. , Seyednasrollah, B. , Perez‐Priego, O. , Gonzalez‐Cascon, R. , Martín, M. , Moreno, G. , Nair, R. , Wutzler, T. , Franziska Bucher, S. , Carrara, A. , Cremonese, E. , EI‐Madany, T. , Filippa, G. , Galvagno, M. , Hammer, T. , Ma, X. , & Migliavacca, M. (2022). Evergreen broadleaf greenness and its relationship with leaf flushing, aging, and water fluxes. Agricultural and Forest Meteorology, 323, 109060. 10.1016/j.agrformet.2022.109060

[gcb16436-bib-0099] Luyssaert, S. , Janssens, I. A. , Sulkava, M. , Papale, D. , Dolman, A. J. , Reichstein, M. , Hollmén, J. , Martin, J. G. , Suni, T. , Vesala, T. , Loustau, D. , Law, B. E. , & Moors, E. J. (2007). Photosynthesis drives anomalies in net carbon‐exchange of pine forests at different latitudes. Global Change Biology, 13(10), 2110–2127. 10.1111/j.1365-2486.2007.01432.x

[gcb16436-bib-0100] Ma, X. , Huete, A. , Tran, N. , Bi, J. , Gao, S. , & Zeng, Y. (2020). Sun‐angle effects on remote‐sensing phenology observed and modelled using Himawari‐8. Remote Sensing, 12(8), 1339. 10.3390/rs12081339

[gcb16436-bib-0101] Ma, X. , Huete, A. , & Tran, N. N. (2019). Interaction of seasonal sun‐angle and Savanna phenology observed and modelled using MODIS. Remote Sensing, 11(12), 1398. 10.3390/rs11121398

[gcb16436-bib-0102] MacKenzie, C. M. , Murray, G. , Primack, R. , & Weihrauch, D. (2017). Lessons from citizen science: Assessing volunteer‐collected plant phenology data with Mountain Watch. Biological Conservation, 208, 121–126. 10.1016/j.biocon.2016.07.027

[gcb16436-bib-0103] Marchin, R. M. , McHugh, I. , Simpson, R. R. , Ingram, L. J. , Balas, D. S. , Evans, B. J. , & Adams, M. A. (2018). Productivity of an Australian mountain grassland is limited by temperature and dryness despite long growing seasons. Agricultural and Forest Meteorology, 256–257, 116–124. 10.1016/j.agrformet.2018.02.030

[gcb16436-bib-0104] McGowan, M. M. , Perlut, N. G. , & Strong, A. M. (2021). Agriculture is adapting to phenological shifts caused by climate change, but grassland songbirds are not. Ecology and Evolution, 11(11), 6993–7002. 10.1002/ece3.7548 34141270 PMC8207150

[gcb16436-bib-0105] McInnes, R. N. , Hemming, D. , Burgess, P. , Lyndsay, D. , Osborne, N. J. , Skjoth, C. A. , Thomas, S. , & Vardoulakis, S. (2017). Mapping allergenic pollen vegetation in UK to study environmental exposure and human health. Science of the Total Environment, 599–600, 483–499. 10.1016/j.scitotenv.2017.04.136 PMC5593151 28482306

[gcb16436-bib-0106] Mehajan, R. K. , & Verma, S. (2020). Prospective applications of artificial intelligence/machine learning techniques in earth sciences. Current Science, 119(3), 424.

[gcb16436-bib-0107] Melaas, E. K. , Friedl, M. A. , & Zhu, Z. (2013). Detecting interannual variation in deciduous broadleaf forest phenology using Landsat TM/ETM+ data. Remote Sensing of Environment, 132, 176–185. 10.1016/j.rse.2013.01.011

[gcb16436-bib-0108] Migliavacca, M. , Galvagno, M. , Cremonese, E. , Rossini, M. , Meroni, M. , Sonnentag, O. , Cogliati, S. , Manca, G. , Diotri, F. , Busetto, L. , Cescatti, A. , Colombo, R. , Fava, F. , Morra di Cella, U. , Pari, E. , Siniscalco, C. , & Richardson, A. D. (2011). Using digital repeat photography and eddy covariance data to model grassland phenology and photosynthetic CO_2_ uptake. Agricultural and Forest Meteorology, 151(10), 1325–1337. 10.1016/j.agrformet.2011.05.012

[gcb16436-bib-0109] Moon, M. , Richardson, A. D. , & Friedl, M. A. (2021). Multiscale assessment of land surface phenology from harmonized Landsat 8 and Sentinel‐2, PlanetScope, and PhenoCam imagery. Remote Sensing of Environment, 266, 112716. 10.1016/j.rse.2021.112716

[gcb16436-bib-0110] Moon, M. , Richardson, A. D. , Milliman, T. , & Friedl, M. A. (2022). A high spatial resolution land surface phenology dataset for AmeriFlux and NEON sites. Scientific Data, 9(1), 448. 10.1038/s41597-022-01570-5 35896546 PMC9329431

[gcb16436-bib-0111] Moore, C. E. , Brown, T. , Keenan, T. F. , Duursma, R. A. , van Dijk, A. I. J. M. , Beringer, J. , Culvenor, D. , Evans, B. , Huete, A. , Hutley, L. B. , Maier, S. , Restrepo‐Coupe, N. , Sonnentag, O. , Specht, A. , Taylor, J. R. , van Gorsel, E. , & Liddell, M. J. (2016). Reviews and syntheses: Australian vegetation phenology: New insights from satellite remote sensing and digital repeat photography. Biogeosciences, 13(17), 5085–5102. 10.5194/bg-13-5085-2016

[gcb16436-bib-0112] Morton, D. C. , Nagol, J. , Carabajal, C. C. , Rosette, J. , Palace, M. , Cook, B. D. , Vermote, E. F. , Harding, D. J. , & North, P. J. (2014). Amazon forests maintain consistent canopy structure and greenness during the dry season. Nature, 506, 221–224. 10.1038/nature13006 24499816

[gcb16436-bib-0113] Munson, S. M. , & Long, A. L. (2017). Climate drives shifts in grass reproductive phenology across the western USA. New Phytologist, 213(4), 1945–1955. 10.1111/nph.14327 27870060

[gcb16436-bib-0114] Nagai, S. , Yasuyuki, M. , Saitoh, T. M. , & Narumasa, T. (2021). Usefulness of social sensing using text mining of tweets for detection of autumn phenology. Frontiers in Forests and Global Change, 4, 659910. 10.3389/ffgc.2021.659910

[gcb16436-bib-0115] Nair, R. K. F. , Morris, K. A. , Hertel, M. , Luo, Y. , Moreno, G. , Reichstein, M. , Schrumpf, M. , & Migliavacca, M. (2019). N:P stoichiometry and habitat effects on Mediterranean savanna seasonal root dynamics. Biogeosciences, 16(9), 1883–1901. 10.5194/bg-16-1883-2019

[gcb16436-bib-0116] Nasahara, K. N. , & Nagai, S. (2015). Review: Development of an in situ observation network for terrestrial ecological remote sensing: The Phenological Eyes Network (PEN). Ecological Research, 30(2), 211–223. 10.1007/s11284-014-1239-x

[gcb16436-bib-0117] Nietupski, T. C. , Kennedy, R. E. , Temesgen, H. , & Kerns, B. K. (2021). Spatiotemporal image fusion in Google Earth Engine for annual estimates of land surface phenology in a heterogenous landscape. International Journal of Applied Earth Observation and Geoinformation, 99, 102323. 10.1016/j.jag.2021.102323

[gcb16436-bib-0118] Norby, R. J. (2021). Comment on “Increased growing‐season productivity drives earlier autumn leaf senescence in temperate trees”. Science, 371, 6533. 10.1126/science.abg1438 33674465

[gcb16436-bib-0119] Norris, J. R. , & Walker, J. J. (2020). Solar and sensor geometry, not vegetation response, drive satellite NDVI phenology in widespread ecosystems of the western United States. Remote Sensing of Environment, 249, 112013. 10.1016/j.rse.2020.112013

[gcb16436-bib-0120] O'Connell, J. L. , & Alber, M. (2016). A smart classifier for extracting environmental data from digital image time‐series: Applications for PhenoCam data in a tidal salt marsh. Environmental Modelling & Software, 84, 134–139. 10.1016/j.envsoft.2016.06.025

[gcb16436-bib-0121] Peng, D. , Zhang, X. , Wu, C. , Huang, W. , Gonsamo, A. , Huete, A. R. , Didan, K. , Tan, B. , Liu, X. , & Zhang, B. (2017). Intercomparison and evaluation of spring phenology products using National Phenology Network and AmeriFlux observations in the contiguous United States. Agricultural and Forest Meteorology, 242, 33–46. 10.1016/j.agrformet.2017.04.009

[gcb16436-bib-0122] Peng, D. , Zhang, X. , Zhang, B. , Liu, L. , Liu, X. , Huete, A. R. , Huang, W. , Wang, S. , Luo, S. , Zhang, X. , & Zhang, H. (2017). Scaling effects on spring phenology detections from MODIS data at multiple spatial resolutions over the contiguous United States. ISPRS Journal of Photogrammetry and Remote Sensing, 132, 185–198. 10.1016/j.isprsjprs.2017.09.002

[gcb16436-bib-0123] Petri, C. A. , & Galvão, L. S. (2019). Sensitivity of seven MODIS vegetation indices to BRDF effects during the Amazonian dry season. Remote Sensing, 11(14), 1650. 10.3390/rs11141650

[gcb16436-bib-0124] Piao, S. , Friedlingstein, P. , Ciais, P. , Viovy, N. , & Demarty, J. (2007). Growing season extension and its impact on terrestrial carbon cycle in the Northern Hemisphere over the past 2 decades. Global Biogeochemical Cycles, 21(3), GB3018. 10.1029/2006GB002888

[gcb16436-bib-0125] Piao, S. , Liu, Q. , Chen, A. , Janssens, I. A. , Fu, Y. , Dai, J. , Liu, L. , Lian, X. , Shen, M. , & Zhu, X. (2019). Plant phenology and global climate change: Current progresses and challenges. Global Change Biology, 25(6), 1922–1940. 10.1111/gcb.14619 30884039

[gcb16436-bib-0126] Piao, S. , Tan, J. , Chen, A. , Fu, Y. H. , Ciais, P. , Liu, Q. , Janssens, I. A. , Vicca, S. , Zeng, Z. , Jeong, S. J. , Li, Y. , Myneni, R. B. , Peng, S. , Shen, M. , & Penuelas, J. (2015). Leaf onset in the northern hemisphere triggered by daytime temperature. Nature Communications, 6, 6911. 10.1038/ncomms7911 PMC4423217 25903224

[gcb16436-bib-0127] Potgieter, A. B. , George‐Jaeggli, B. , Chapman, S. C. , Laws, K. , Suárez, C. L. A. , Wixted, J. , Watson, J. , Eldridge, M. , Jordan, D. R. , & Hammer, G. L. (2017). Multi‐spectral imaging from an unmanned aerial vehicle enables the assessment of seasonal leaf area dynamics of sorghum breeding lines. Frontiers in Plant Science, 8, 1532. 10.3389/fpls.2017.01532 28951735 PMC5599772

[gcb16436-bib-0128] Qiu, T. , Song, C. , & Li, J. (2017). Impacts of urbanization on vegetation phenology over the past three decades in Shanghai, China. Remote Sensing, 9(9), 970. 10.3390/rs9090970

[gcb16436-bib-0129] Ren, S. , & Peichl, M. (2021). Enhanced spatiotemporal heterogeneity and the climatic and biotic controls of autumn phenology in northern grasslands. Science of the Total Environment, 788, 147806. 10.1016/j.scitotenv.2021.147806 34029811

[gcb16436-bib-0130] Ren, S. , Qin, Q. , & Ren, H. (2019). Contrasting wheat phenological responses to climate change in global scale. Science of the Total Environment, 665, 620–631. 10.1016/j.scitotenv.2019.01.394 30776634

[gcb16436-bib-0131] Reyes‐Fox, M. , Steltzer, H. , Trlica, M. J. , McMaster, G. S. , Andales, A. A. , LeCain, D. R. , & Morgan, J. A. (2014). Elevated CO_2_ further lengthens growing season under warming conditions. Nature, 510(7504), 259–262. 10.1038/nature13207 24759322

[gcb16436-bib-0132] Reynolds, J. F. , Kemp, P. R. , Ogle, K. , & Fernández, R. J. (2004). Modifying the ‘pulse–reserve’ paradigm for deserts of North America: Precipitation pulses, soil water, and plant responses. Oecologia, 141(2), 194–210. 10.1007/s00442-004-1524-4 15042457

[gcb16436-bib-0133] Richardson, A. D. (2019). Tracking seasonal rhythms of plants in diverse ecosystems with digital camera imagery. New Phytologist, 222(4), 1742–1750. 10.1111/nph.15591 30415486

[gcb16436-bib-0134] Richardson, A. D. , Anderson, R. S. , Arain, M. A. , Barr, A. G. , Bohrer, G. , Chen, G. , Chen, J. M. , Ciais, P. , Davis, K. J. , Desai, A. R. , Dietze, M. C. , Dragoni, D. , Garrity, S. R. , Gough, C. M. , Grant, R. , Hollinger, D. Y. , Margolis, H. A. , McCaughey, H. , Migliavacca, M. , … Xue, Y. (2012). Terrestrial biosphere models need better representation of vegetation phenology: Results from the North American Carbon Program Site Synthesis. Global Change Biology, 18(2), 566–584. 10.1111/j.1365-2486.2011.02562.x

[gcb16436-bib-0135] Richardson, A. D. , Black, T. A. , Ciais, P. , Delbart, N. , Friedl, M. A. , Gobron, N. , Hollinger, D. Y. , Kutsch, W. L. , Longdoz, B. , Luyssaert, S. , Migliavacca, M. , Montagnani, L. , Munger, J. W. , Moors, E. , Piao, S. , Rebmann, C. , Reichstein, M. , Saigusa, N. , Tomelleri, E. , … Varlagin, A. (2010). Influence of spring and autumn phenological transitions on forest ecosystem productivity. Philosophical Transactions of the Royal Society B: Biological Sciences, 365(1555), 3227–3246. 10.1098/rstb.2010.0102 PMC2981939 20819815

[gcb16436-bib-0136] Richardson, A. D. , Hufkens, K. , Milliman, T. , Aubrecht, D. M. , Chen, M. , Gray, J. M. , Johnston, M. R. , Keenan, T. F. , Klosterman, S. T. , Kosmala, M. , Melaas, E. K. , Friedl, M. A. , & Frolking, S. (2018). Tracking vegetation phenology across diverse North American biomes using PhenoCam imagery. Scientific Data, 5, 180028. 10.1038/sdata.2018.28 29533393 PMC5848786

[gcb16436-bib-0137] Richardson, A. D. , Hufkens, K. , Milliman, T. , Aubrecht, D. M. , Furze, M. E. , Seyednasrollah, B. , Krassovski, M. , Latimer, J. , Robert Nettles, W. , Heiderman, R. , Warren, J. , & Hanson, P. (2018). Ecosystem warming extends vegetation activity but heightens vulnerability to cold temperatures. Nature, 560(7718), 368–371. 10.1038/s41586-018-0399-1 30089905

[gcb16436-bib-0138] Richardson, A. D. , Keenan, T. F. , Migliavacca, M. , Ryu, Y. , Sonnentag, O. , & Toomey, M. (2013). Climate change, phenology, and phenological control of vegetation feedbacks to the climate system. Agricultural and Forest Meteorology, 169, 156–173. 10.1016/j.agrformet.2012.09.012

[gcb16436-bib-0139] Rojo, J. , Rapp, A. , Lara, B. , Fernandez‐Gonzalez, F. , & Perez‐Badia, R. (2015). Effect of land uses and wind direction on the contribution of local sources to airborne pollen. Science of the Total Environment, 538, 672–682. 10.1016/j.scitotenv.2015.08.074 26327635

[gcb16436-bib-0140] Sakamoto, T. , Gitelson, A. A. , Nguy‐Robertson, A. L. , Arkebauer, T. J. , Wardlow, B. D. , Suyker, A. E. , Verma, S. B. , & Shibayama, M. (2012). An alternative method using digital cameras for continuous monitoring of crop status. Agricultural and Forest Meteorology, 154–155, 113–126. 10.1016/j.agrformet.2011.10.014

[gcb16436-bib-0141] Sánchez, J. M. , Aznarte, M. J. L. , Lugilde, D. N. , Fernández, C. L. , Guardia, C. D. , & AlbaSánchez, F. (2007). Forecasting airborne pollen concentration time series with neural and neuro‐fuzzy models. Expert Systems with Applications, 32(4), 1218–1225. 10.1016/j.eswa.2006.02.011

[gcb16436-bib-0142] Schaaf, C. B. , Gao, F. , Strahler, A. H. , Lucht, W. , Li, X. , Tsang, T. , Strugnell, N. C. , Zhang, X. , Jin, Y. , Muller, J. P. , Lewis, P. , Barnsley, M. , Hobson, P. , Hobson, M. , Roberts, G. , Dunderdale, M. , Doll, C. , d'Entremont, R. P. , Hu, B. , … Roy, D. (2002). First operational BRDF, albedo nadir reflectance products from MODIS. Remote Sensing of Environment, 83, 135–148. 10.1016/S0034-4257(02)00091-3

[gcb16436-bib-0143] Seyednasrollah, B. , Young, A. M. , Hufkens, K. , Milliman, T. , Friedl, M. A. , Frolking, S. , & Richardson, A. D. (2019). Tracking vegetation phenology across diverse biomes using Version 2.0 of the PhenoCam Dataset. Scientific Data, 6(1), 222. 10.1038/s41597-019-0229-9 31641140 PMC6805894

[gcb16436-bib-0144] Shen, M. , Zhang, G. , Cong, N. , Wang, S. , Kong, W. , & Piao, S. (2014). Increasing altitudinal gradient of spring vegetation phenology during the last decade on the Qinghai–Tibetan Plateau. Agricultural and Forest Meteorology, 189–190, 71–80. 10.1016/j.agrformet.2014.01.003

[gcb16436-bib-0145] Shi, P. , Chen, Z. , Reddy, G. V. P. , Hui, C. , Huang, J. , & Xiao, M. (2017). Timing of cherry tree blooming: Contrasting effects of rising winter low temperatures and early spring temperatures. Agricultural and Forest Meteorology, 240, 78–89. 10.1016/j.agrformet.2017.04.001

[gcb16436-bib-0146] Shu, M. , Shen, M. , Dong, Q. , Yang, X. , Li, B. , & Ma, Y. (2022). Estimating the maize above‐ground biomass by constructing the tridimensional concept model based on UAV‐based digital and multi‐spectral images. Field Crops Research, 282, 108491. 10.1016/j.fcr.2022.108491

[gcb16436-bib-0147] Silver, J. D. , Spriggs, K. , Haberle, S. G. , Katelaris, C. H. , Newbigin, E. J. , & Lampugnani, E. R. (2020). Using crowd‐sourced allergic rhinitis symptom data to improve grass pollen forecasts and predict individual symptoms. Science of the Total Environment, 720, 137351. 10.1016/j.scitotenv.2020.137351 32325552

[gcb16436-bib-0148] Skjøth, C. A. , Ørby, P. V. , Becker, T. , Geels, C. , Schlünssen, V. , Sigsgaard, T. , Bønløkke, J. H. , Sommer, J. , Søgaard, P. , & Hertel, O. (2013). Identifying urban sources as cause of elevated grass pollen concentrations using GIS and remote sensing. Biogeosciences, 10(1), 541–554. 10.5194/bg-10-541-2013

[gcb16436-bib-0149] Skjøth, C. A. , Smith, M. , Šikoparija, B. , Stach, A. , Myszkowska, D. , Kasprzyk, I. , Radišić, P. , Stjepanović, B. , Hrga, I. , & Apatini, D. (2010). A method for producing airborne pollen source inventories: An example of Ambrosia (ragweed) on the Pannonian Plain. Agricultural and Forest Meteorology, 150(9), 1203–1210. 10.1016/j.agrformet.2010.05.002

[gcb16436-bib-0150] Smith, M. , & Emberlin, J. (2006). A 30‐day‐ahead forecast model for grass pollen in north London, United Kingdom. International Journal of Biometeorology, 50(4), 233–242. 10.1007/s00484-005-0010-y 16391931

[gcb16436-bib-0151] Song, G. , Wu, S. , Lee, C. K. F. , Serbin, S. P. , Wolfe, B. T. , Ng, M. K. , Ely, K. S. , Bogonovich, M. , Wang, J. , Lin, Z. , Saleska, S. , Nelson, B. W. , Rogers, A. , & Wu, J. (2022). Monitoring leaf phenology in moist tropical forests by applying a superpixel‐based deep learning method to time‐series images of tree canopies. ISPRS Journal of Photogrammetry and Remote Sensing, 183, 19–33. 10.1016/j.isprsjprs.2021.10.023

[gcb16436-bib-0152] Songsom, V. , Koedsin, W. , Ritchie, R. J. , & Huete, A. (2021). Mangrove phenology and water influences measured with digital repeat photography. Remote Sensing, 13(2), 307. 10.3390/rs13020307

[gcb16436-bib-0153] Sonnentag, O. , Detto, M. , Vargas, R. , Ryu, Y. , Runkle, B. R. K. , Kelly, M. , & Baldocchi, D. D. (2011). Tracking the structural and functional development of a perennial pepperweed (*Lepidium latifolium* L.) infestation using a multi‐year archive of webcam imagery and eddy covariance measurements. Agricultural and Forest Meteorology, 151(7), 916–926. 10.1016/j.agrformet.2011.02.011

[gcb16436-bib-0154] Sonnentag, O. , Hufkens, K. , Teshera‐Sterne, C. , Young, A. M. , Friedl, M. , Braswell, B. H. , Milliman, T. , O'Keefe, J. , & Richardson, A. D. (2012). Digital repeat photography for phenological research in forest ecosystems. Agricultural and Forest Meteorology, 152, 159–177. 10.1016/j.agrformet.2011.09.009

[gcb16436-bib-0155] Su, J. , Liu, C. , Coombes, M. , Hu, X. , Wang, C. , Xu, X. , Li, Q. , Guo, L. , & Chen, H. (2018). Wheat yellow rust monitoring by learning from multispectral UAV aerial imagery. Computers and Electronics in Agriculture, 155, 157–166. 10.1016/j.compag.2018.10.017

[gcb16436-bib-0156] Sun, Z. , Sandoval, L. , Crystal‐Ornelas, R. , Mousavi, S. M. , Wang, J. , Lin, C. , Cristea, N. , Tong, D. , Carande, W. H. , Ma, X. , Rao, Y. , Bednar, J. A. , Tan, A. , Wang, J. , Purushotham, S. , Gill, T. E. , Chastang, J. , Howard, D. , Holt, B. , … John, A. (2022). A review of Earth Artificial Intelligence. Computers and Geosciences, 159, 105034. 10.1016/j.cageo.2022.105034

[gcb16436-bib-0157] Tang, J. , Körner, C. , Muraoka, H. , Piao, S. , Shen, M. , Thackeray, S. J. , & Yang, X. (2016). Emerging opportunities and challenges in phenology: A review. Ecosphere, 7(8), 01436. 10.1002/ecs2.1436

[gcb16436-bib-0158] Tang, X. , Li, H. , Desai, A. R. , Nagy, Z. , Luo, J. , Kolb, T. E. , Olioso, A. , Xu, X. , Yao, L. , Kutsch, W. , Pilegaard, K. , Kostner, B. , & Ammann, C. (2014). How is water‐use efficiency of terrestrial ecosystems distributed and changing on Earth? Scientific Reports, 4, 7483. 10.1038/srep07483 25500908 PMC4265788

[gcb16436-bib-0159] Thapa, S. , Garcia Millan, V. E. , & Eklundh, L. (2021). Assessing forest phenology: A multi‐scale comparison of near‐surface (UAV, spectral reflectance sensor, PhenoCam) and satellite (MODIS, Sentinel‐2) remote sensing. Remote Sensing, 13(8), 1597. 10.3390/rs13081597

[gcb16436-bib-0160] Thibaudon, M. , Šikoparija, B. , Oliver, G. , Smith, M. , & Skjøth, C. A. (2014). Ragweed pollen source inventory for France—The second largest centre of Ambrosia in Europe. Atmospheric Environment, 83, 62–71. 10.1016/j.atmosenv.2013.10.057

[gcb16436-bib-0161] Tian, F. , Cai, Z. , Jin, H. , Hufkens, K. , Scheifinger, H. , Tagesson, T. , Smets, B. , Van Hoolst, R. , Bonte, K. , Ivits, E. , Tong, X. , Ardö, J. , & Eklundh, L. (2021). Calibrating vegetation phenology from Sentinel‐2 using eddy covariance, PhenoCam, and PEP725 networks across Europe. Remote Sensing of Environment, 260, 112456. 10.1016/j.rse.2021.112456

[gcb16436-bib-0162] Tian, J. , Zhu, X. , Chen, J. , Wang, C. , Shen, M. , Yang, W. , Tan, X. , Xu, S. , & Li, Z. (2021). Improving the accuracy of spring phenology detection by optimally smoothing satellite vegetation index time series based on local cloud frequency. ISPRS Journal of Photogrammetry and Remote Sensing, 180, 29–44. 10.1016/j.isprsjprs.2021.08.003

[gcb16436-bib-0163] Tian, J. , Zhu, X. , Shen, Z. , Wu, J. , Xu, S. , Liang, Z. , & Wang, J. (2020). Investigating the urban‐induced microclimate effects on winter wheat spring phenology using Sentinel‐2 time series. Agricultural and Forest Meteorology, 294, 108153. 10.1016/j.agrformet.2020.108153

[gcb16436-bib-0164] Tian, J. , Zhu, X. , Wan, L. , & Collin, M. (2021). Impacts of satellite revisit frequency on spring phenology monitoring of deciduous broad‐leaved forests based on vegetation index time series. IEEE Journal of Selected Topics in Applied Earth Observations and Remote Sensing, 14, 10500–10508. 10.1109/JSTARS.2021.3120013

[gcb16436-bib-0165] Tian, J. , Zhu, X. , Wu, J. , Shen, M. , & Chen, J. (2020). Coarse‐resolution satellite images overestimate urbanization effects on vegetation spring phenology. Remote Sensing, 12(1), 117. 10.3390/rs12010117

[gcb16436-bib-0166] Toomey, M. , Friedl, M. A. , Frolking, S. , Hufkens, K. , Klosterman, S. , Sonnentag, O. , Baldocchi, D. D. , Bernacchi, C. J. , Biraud, S. C. , Bohrer, G. , Brzostek, E. , Burns, S. P. , Coursolle, C. , Hollinger, D. Y. , Margolis, H. A. , McCaughey, H. , Monson, R. K. , Munger, J. W. , Pallardy, S. , … Richardson, A. D. (2015). Greenness indices from digital cameras predict the timing and seasonal dynamics of canopy‐scale photosynthesis. Ecological Applications, 25(1), 99–115. 10.1890/14-0005.1 26255360

[gcb16436-bib-0167] Utz, R. M. , & Prism, J. A. (2012). The national ecological observatory network: A large facility observatory poised to expand spatiotemporal scales of inquiry in fisheries science. Fisheries, 38, 26–35. 10.1080/03632415.2013.748551

[gcb16436-bib-0168] Vázquez‐Lule, A. , & Vargas, R. (2021). Biophysical drivers of net ecosystem and methane exchange across phenological phases in a tidal salt marsh. Agricultural and Forest Meteorology, 300, 108309. 10.1016/j.agrformet.2020.108309

[gcb16436-bib-0169] Vitasse, Y. , Baumgarten, F. , Zohner, C. M. , Rutishauser, T. , Pietragalla, B. , Gehrig, R. , Dai, J. , Wang, H. , Aono, Y. , & Sparks, T. (2022). The great acceleration of plant phenological shifts. Nature Climate Change, 12(4), 300–302. 10.1038/s41558-022-01283-y

[gcb16436-bib-0170] Voukantsis, D. , Niska, H. , Karatzas, K. , Riga, M. , Damialis, A. , & Vokou, D. (2010). Forecasting daily pollen concentrations using data‐driven modeling methods in Thessaloniki, Greece. Atmospheric Environment, 44(39), 5101–5111. 10.1016/j.atmosenv.2010.09.006

[gcb16436-bib-0171] Wallace, C. , Walker, J. , Skirvin, S. , Patrick‐Birdwell, C. , Weltzin, J. , & Raichle, H. (2016). Mapping presence and predicting phenological status of invasive buffelgrass in Southern Arizona using MODIS, climate and citizen science observation data. Remote Sensing, 8(7), 524. 10.3390/rs8070524

[gcb16436-bib-0172] Wang, C. , Wu, Y. , Hu, Q. , Hu, J. , Chen, Y. , Lin, S. , & Xie, Q. (2022). Comparison of vegetation phenology derived from solar‐induced chlorophyll fluorescence and Enhanced Vegetation Index, and their relationship with climate limitations. Remote Sensing, 14(13), 3018. 10.3390/rs14133018

[gcb16436-bib-0173] Wang, M. , Li, P. , Peng, C. , Xiao, J. , Zhou, X. , Luo, Y. , & Zhang, C. (2022). Divergent responses of autumn vegetation phenology to climate extremes over northern middle and high latitudes. Global Ecology and Biogeography, 1–16. 10.1111/geb.13583

[gcb16436-bib-0174] Wang, X. , Wu, C. , Zhang, X. , Li, Z. , Liu, Z. , Gonsamo, A. , & Ge, Q. (2020). Satellite‐observed decrease in the sensitivity of spring phenology to climate change under high nitrogen deposition. Environmental Research Letters, 15(9), 094055. 10.1088/1748-9326/aba57f

[gcb16436-bib-0175] Wilson, A. M. , & Jetz, W. (2016). Remotely sensed high‐resolution global cloud dynamics for predicting ecosystem and biodiversity distributions. PLoS Biology, 14(3), 1002415. 10.1371/journal.pbio.1002415 PMC4816575 27031693

[gcb16436-bib-0176] Wingate, L. , Ogée, J. , Cremonese, E. , Filippa, G. , Mizunuma, T. , Migliavacca, M. , Moisy, C. , Wilkinson, M. , Moureaux, C. , Wohlfahrt, G. , Hammerle, A. , Hörtnagl, L. , Gimeno, C. , Porcar‐Castell, A. , Galvagno, M. , Nakaji, T. , Morison, J. , Kolle, O. , Knohl, A. , … Grace, J. (2015). Interpreting canopy development and physiology using a European phenology camera network at flux sites. Biogeosciences, 12(20), 5995–6015. 10.5194/bg-12-5995-2015

[gcb16436-bib-0177] WMO . (2022). State of the global climate . https://library.wmo.int/doc_num.php?explnum_id=11178

[gcb16436-bib-0178] Woebbecke, D. M. , Meyer, G. E. , Bargen, K. V. , & Mortensen, D. A. J. (1995). Color indices for weed identification under various soil, residue, and lighting conditions. American Society of Agricultural Engineers, 38(1), 259–269.

[gcb16436-bib-0179] Wolf, S. , Keenan, T. F. , Fisher, J. B. , Baldocchi, D. D. , Desai, A. R. , Richardson, A. D. , Scott, R. L. , Law, B. E. , Litvak, M. E. , Brunsell, N. A. , Peters, W. , & van der Laan‐Luijkx, I. T. (2016). Warm spring reduced carbon cycle impact of the 2012 US summer drought. Proceedings of the National Academy of Sciences of the United States of America, 113(21), 5880–5885. 10.1073/pnas.1519620113 27114518 PMC4889356

[gcb16436-bib-0180] Wu, C. , Chen, J. M. , Black, T. A. , Price, D. T. , Kurz, W. A. , Desai, A. R. , Gonsamo, A. , Jassal, R. S. , Gough, C. M. , Bohrer, G. , Dragoni, D. , Herbst, M. , Gielen, B. , Berninger, F. , Vesala, T. , Mammarella, I. , Pilegaard, K. , & Blanken, P. D. (2013). Interannual variability of net ecosystem productivity in forests is explained by carbon flux phenology in autumn. Global Ecology and Biogeography, 22(8), 994–1006. 10.1111/geb.12044

[gcb16436-bib-0181] Wu, J. , Albert, L.P. , Lopes, A.P. , Restrepo‐Coupe, N. , Hayek, M. , Wiedemann, K.T. , Guan, K. , Stark, S.C. , Christoffersen, B. , Prohaska, N. , Tavares, J.V. , Marostica, S. , Kobayashi, H. , L. Ferreira , M., Campos, K.S. , Silva, R.d ., Brando, P.M. , Dye, D.G. , Huxman, T.E. , Huete, A.R. , Nelson, B.W. , & Saleska, S.R . (2016). Leaf development and demography explain photosynthetic seasonality in Amazon evergreen forests. Science, 351(6276), 972‐976. 10.1126/science.aad5068 26917771

[gcb16436-bib-0182] Wu, J. , Kobayashi, H. , Stark, S. C. , Meng, R. , Guan, K. , Tran, N. N. , Gao, S. , Yang, W. , Restrepo‐Coupe, N. , Miura, T. , Oliviera, R. C. , Rogers, A. , Dye, D. G. , Nelson, B. W. , Serbin, S. P. , Huete, A. R. , & Saleska, S. R. (2018). Biological processes dominate seasonality of remotely sensed canopy greenness in an Amazon evergreen forest. New Phytologist, 217(4), 1507–1520. 10.1111/nph.14939 29274288

[gcb16436-bib-0183] Xie, Q. , Cleverly, J. , Moore, C. E. , Ding, Y. , Hall, C. C. , Ma, X. , Brown, L. A. , Wang, C. , Beringer, J. , Prober, S. M. , Macfarlane, C. , Meyer, W. S. , Yin, G. , & Huete, A. (2022). Land surface phenology retrievals for arid and semi‐arid ecosystems. ISPRS Journal of Photogrammetry and Remote Sensing, 185, 129–145. 10.1016/j.isprsjprs.2022.01.017

[gcb16436-bib-0184] Zani, D. , Crowther, T. W. , Mo, L. , Renner, S. S. , & Zohner, C. M. (2020). Increased growing‐season productivity drives earlier autumn leaf senescence in temperate trees. Science, 370(6520), 1066–1071. 10.1126/science.abd8911 33243884

[gcb16436-bib-0185] Zeng, L. , Wardlow, B. D. , Xiang, D. , Hu, S. , & Li, D. (2020). A review of vegetation phenological metrics extraction using time‐series, multispectral satellite data. Remote Sensing of Environment, 237, 111511. 10.1016/j.rse.2019.111511

[gcb16436-bib-0186] Zha, T. , Barr, A. G. , van der Kamp, G. , Black, T. A. , McCaughey, J. H. , & Flanagan, L. B. (2010). Interannual variation of evapotranspiration from forest and grassland ecosystems in western Canada in relation to drought. Agricultural and Forest Meteorology, 150(11), 1476–1484. 10.1016/j.agrformet.2010.08.003

[gcb16436-bib-0187] Zhang, X. , Friedl, M. A. , & Schaaf, C. B. (2009). Sensitivity of vegetation phenology detection to the temporal resolution of satellite data. International Journal of Remote Sensing, 30(8), 2061–2074. 10.1080/01431160802549237

[gcb16436-bib-0188] Zhang, X. , Friedl, M. A. , Schaaf, C. B. , Strahler, A. H. , Hodges, J. C. F. , Gao, F. , Reed, B. C. , & Huete, A. (2003). Monitoring vegetation phenology using MODIS. Remote Sensing of Environment, 84, 471–475. 10.1016/S0034-4257(02)00135-9

[gcb16436-bib-0189] Zhang, X. , Jayavelu, S. , Liu, L. , Friedl, M. A. , Henebry, G. M. , Liu, Y. , Schaaf, C. B. , Richardson, A. D. , & Gray, J. (2018). Evaluation of land surface phenology from VIIRS data using time series of PhenoCam imagery. Agricultural and Forest Meteorology, 256–257, 137–149. 10.1016/j.agrformet.2018.03.003

[gcb16436-bib-0190] Zhang, X. , Wang, J. , Gao, F. , Liu, Y. , Schaaf, C. , Friedl, M. , Yu, Y. , Jayavelu, S. , Gray, J. , Liu, L. , Yan, D. , & Henebry, G. M. (2017). Exploration of scaling effects on coarse resolution land surface phenology. Remote Sensing of Environment, 190, 318–330. 10.1016/j.rse.2017.01.001

[gcb16436-bib-0191] Zhang, X. , Wang, J. , Henebry, G. M. , & Gao, F. (2020). Development and evaluation of a new algorithm for detecting 30 m land surface phenology from VIIRS and HLS time series. ISPRS Journal of Photogrammetry and Remote Sensing, 161, 37–51. 10.1016/j.isprsjprs.2020.01.012

[gcb16436-bib-0192] Zheng, J. , Jia, G. , & Xu, X. (2022). Earlier snowmelt predominates advanced spring vegetation greenup in Alaska. Agricultural and Forest Meteorology, 315, 108828. 10.1016/j.agrformet.2022.108828

[gcb16436-bib-0193] Zheng, Z. , & Zhu, W. (2017). Uncertainty of remote sensing data in monitoring vegetation phenology: A comparison of MODIS C_5_ and C_6_ vegetation index products on the Tibetan Plateau. Remote Sensing, 9(12), 1288. 10.3390/rs9121288

[gcb16436-bib-0194] Zhou, C. , Hua, Y. , Ma, T. , & Pei, T. (2022). On holo‐spatial information system. In New thinking in GIScience (pp. 9–16). Springer. 10.1007/978-981-19-3816-0_2

[gcb16436-bib-0195] Zhou, X. , Zheng, H. B. , Xu, X. Q. , He, J. Y. , Ge, X. K. , Yao, X. , Cheng, T. , Zhu, Y. , Cao, W. X. , & Tian, Y. C. (2017). Predicting grain yield in rice using multi‐temporal vegetation indices from UAV‐based multispectral and digital imagery. ISPRS Journal of Photogrammetry and Remote Sensing, 130, 246–255. 10.1016/j.isprsjprs.2017.05.003

[gcb16436-bib-0196] Zhu, X. , Helmer, E. H. , Gwenzi, D. , Collin, M. , Fleming, S. , Tian, J. , Marcano‐Vega, H. , Meléndez‐Ackerman, E. J. , & Zimmerman, J. K. (2021). Characterization of dry‐season phenology in tropical forests by reconstructing cloud‐free Landsat time series. Remote Sensing, 13(23), 4736. 10.3390/rs13234736

[gcb16436-bib-0197] Zhu, X. , & Liu, D. (2019). Investigating the impact of land parcelization on forest composition and structure in Southeastern Ohio using multi‐source remotely sensed data. Remote Sensing, 11(19), 2195. 10.3390/rs11192195

[gcb16436-bib-0198] Zhu, Y. , Zhang, Y. , Zu, J. , Wang, Z. , Huang, K. , Cong, N. , & Tang, Z. (2019). Effects of data temporal resolution on phenology extractions from the alpine grasslands of the Tibetan Plateau. Ecological Indicators, 104, 365–377. 10.1016/j.ecolind.2019.05.004

[gcb16436-bib-0199] Zohner, C. M. , Mo, L. , Pugh, T. A. M. , Bastin, J. F. , & Crowther, T. W. (2020). Interactive climate factors restrict future increases in spring productivity of temperate and boreal trees. Global Change Biology, 26(7), 4042–4055. 10.1111/gcb.15098 32347650

